# Integrated MCDM framework for sustainable pharmacy supplier selection using pioneering criteria with fuzzy TOPSIS SVR and GRA

**DOI:** 10.1038/s41598-025-02975-z

**Published:** 2025-05-31

**Authors:** R. Raveena, S. Umamaheswari

**Affiliations:** Department of Mathematics, School of Advanced Sciences, Vellore Institute of Technology, Chennai, 600127 India

**Keywords:** Grey relational analysis, Interval-valued triangular fuzzy, Pharmaceuticals, Supplier selection, Support vector regression, Sustainability criteria, TOPSIS, Applied mathematics, Statistics

## Abstract

Supplier selection is indispensable across diverse industries, particularly in the pharmaceutical sector, due to its direct impact on health and safety. This underscores the necessity for the timely availability of high-quality products. This research aims to elevate the sustainability of pharmaceutical inventory management by introducing pioneering criteria for supplier evaluation. A groundbreaking methodology is proposed by seamlessly integrating TOPSIS-GRA within a fuzzy environment for sustainable supplier selection. The CFCS algorithm ensures precise defuzzification, addressing uncertainties in data representation. Additionally, SVR improves criteria weighting by capturing complex data patterns. This integrated approach enables a comprehensive supplier assessment, offering valuable insights to pharmaceutical decision-makers. Ultimately, it provides a robust framework for selecting suppliers aligned with sustainability principles and optimizing procurement practices.

## Introduction

**Figure 1:**
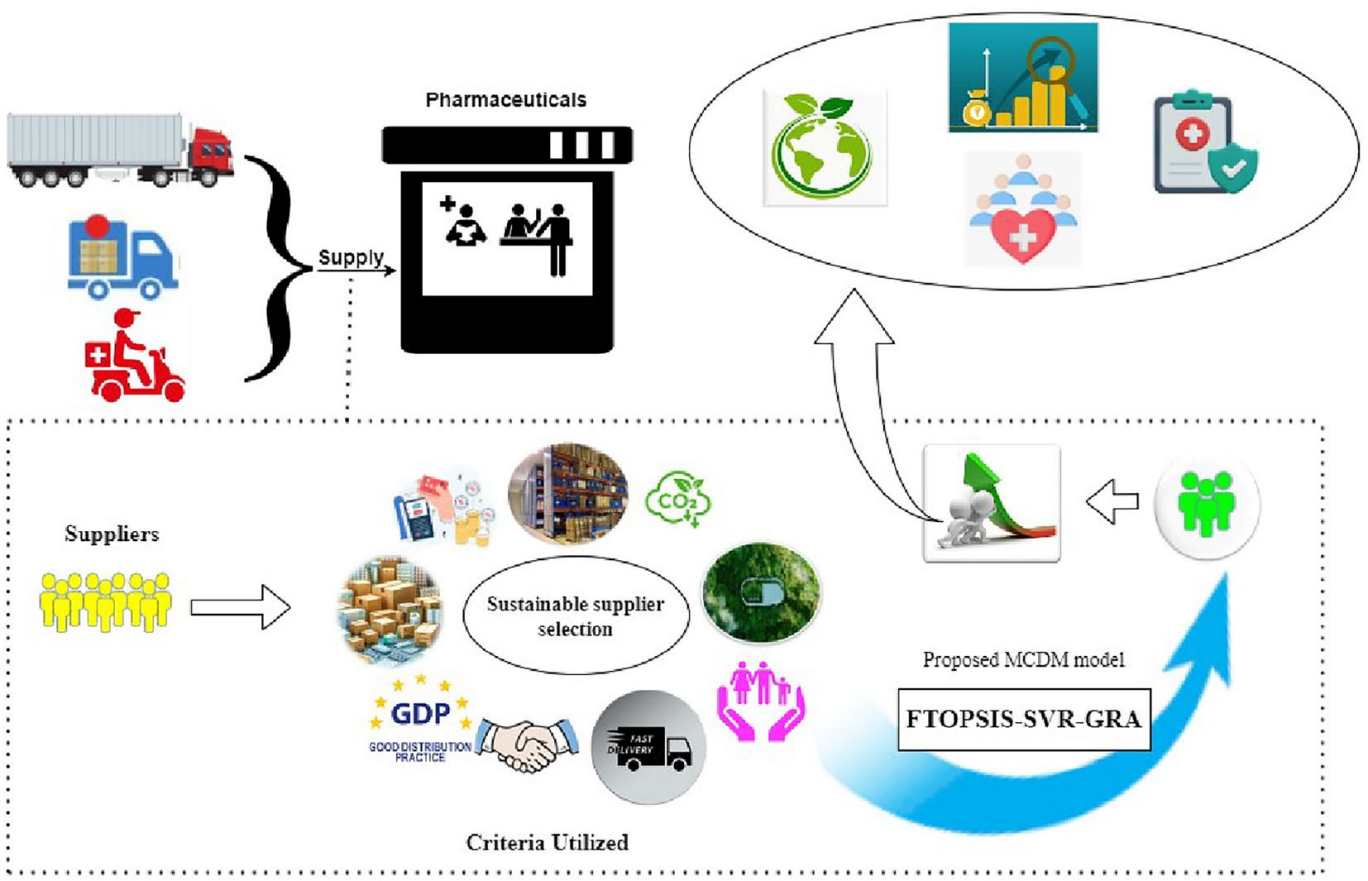
Depiction of the implemented framework.

The healthcare sector is widely recognized as a cornerstone of society due to its role in safeguarding health, promoting stability, and driving economic progress. The multifaceted contributions of pharmaceuticals are constitutive to healthcare, facilitating access to medicines and advancing treatments for various conditions. To ensure proper operational maintenance, excellence in inventory management is essential in pharmaceuticals. This involves stock optimization, waste reduction, profit enhancement, supplier negotiations, cost management, and quality assurance. In the context of inventory management, Umamaheswari et al.,^1^ developed an inventory model to minimize costs for deteriorating items with a positive lead time, using a hyper-geometric distribution to assess outdated costs, particularly for perishable goods with a FIFO policy. Additionally, Umadevi G and Umamaheswari S^2^ emphasized optimizing pharmaceutical inventory to address demand patterns and reduce procurement losses by trend demand and cost matrix analysis. Efficient inventory management relies on well-functioning suppliers. The healthcare sector faces significant challenges, including workforce shortages, rising operational costs, cybersecurity risks^3^, and regulatory requirements. Procurement inefficiencies, data interoperability issues, and increasing demand for personalized care add complexity. Ensuring equitable healthcare access remains a key concern. Effective supplier selection is vital for a stable, cost-efficient, and high-quality medical supply network. In the evolving pharmaceutical landscape, selecting sustainable and reliable suppliers is crucial for maintaining resilience, and ensuring ethical standards. Wholesalers, distributors, and medical suppliers are integral to the pharmaceutical supply, ensuring the efficient and timely delivery of medicines and healthcare. Suppliers are essential for maintaining stock availability, ensuring quality standards, optimizing inventory management, and upholding regulatory compliance. Reliable suppliers ensure the uninterrupted availability of necessary medications, supporting healthcare efficiency and enhancing patient care. Effective supplier selection directly impacts healthcare by enhancing emergency preparedness and maintaining cost efficiency while mitigating the risk of acquiring substandard products. Prioritizing the right suppliers helps sustain efficient operations, minimize supply risks, and enhance patient care by ensuring a steady flow of essential medications. Thus, pharmaceutical suppliers conflict in reliability, efficiency, and value-added benefits such as discounts, extended credit periods, and flexible negotiations. Challenges like supply chain disruptions, fluctuating prices, evolving healthcare policies, regulatory constraints, and inconsistent product quality further complicate the selection process, which emphasizes the need for a robust supplier selection process. Given these complexities, choosing the most suitable supplier requires a structured evaluation. Hence, this study proposes a decision-making model to assess and prioritize suppliers based on critical performance factors, ensuring a resilient and cost-effective supply chain. However, uncertainty and imprecision are inevitable in real-world decision-making, especially when assessing qualitative factors. To address these complexities, Zadeh^4^ introduced the fuzzy set theory, which incorporates elements of fuzziness and allows for the representation of uncertainty through ordered pairs of membership functions. It also provides a more adaptable and effective way to manage uncertainty in decision-making situations. With continuous advancements, fuzzy sets have developed into more refined extensions. Building on these developments, Gorzalczany^5^ introduced IVFS, which enhance traditional fuzzy sets by representing uncertainty through interval-based membership values. This approach is incorporated in Multi-criteria decision-making (MCDM) by providing greater flexibility in handling subjective judgments and complex decision variables. MCDM problems are prevalent across various fields, as it assists decision-makers in assessing and selecting the most suitable alternatives based on multiple conflicting factors. The methodology offers a systematic framework for handling complex decision problems, ensuring a structured evaluation. Recent studies have extensively explored MCDM models, particularly in supplier selection and medical decision-making. Its applications extend to healthcare, robotics^6^, risk analysis, talent optimization^7^, fitness training^8^, manufacturing, and logistics. Over the decades, researchers have explored various MCDM models, with the Technique for Order Preference by Similarity to Ideal Solution (TOPSIS) emerging as one of the effective techniques. Hwang and Yoon^9^ introduced TOPSIS to evaluate alternatives based on multiple factors. FTOPSIS extends TOPSIS by incorporating fuzzy logic, allowing for better handling of ambiguous data. It evaluates alternatives based on their closeness to the fuzzy ideal solution, making it particularly effective in complex environments. Recent refinements have further expanded its applications across various fields, as highlighted in the literature. Grey Relational Analysis (GRA) is integrated with FTOPSIS to provide distinct advantages in decision-making. GRA quantifies the relational closeness between alternatives, refining FTOPSIS rankings by improving the differentiation between closely ranked options. A hybrid FAHP-GRA-TOPSIS model integrates FAHP for criteria weighting and GRA-TOPSIS for ranking, offering a structured and systematic approach to decision-making^10^. The machine learning technique, Support Vector Regression (SVR)^11^ is implemented during decision-making due to its handling of high-dimensional data. It strengthens MCDM by precisely modeling non-linear relationships between alternatives and criteria. This methodology proves particularly beneficial in pharmacy settings, where decisions often involve evaluating suppliers or medication options across multiple criteria. By accommodating uncertainties and imprecision inherent in decision-making, Fuzzy TOPSIS empowers decision-makers in pharmacy to navigate complex scenarios with clarity and precision. With this method, pharmacies can improve the decision-making process, choose the most suitable suppliers, optimize inventory management, ensure the highest standards of product quality, supplier reliability, and cost-effectiveness, and boost operational resilience and sustainability. Ultimately, the proposed model enhances both patient care and operational efficiency in the pharmaceutical environment, and Fig. 1 presents a visual representation.

## Review of literature

Fuzzy decision-making models have been widely applied in pharmaceutical supplier evaluation. A model^12^ integrating QFD, FST, and AHP translates linguistic preferences into a triangular fuzzy matrix, handling qualitative and uncertain criteria for reliable supplier assessment. A study on an Iranian pharmaceutical supply chain applied Fuzzy TOPSIS and PROMETHEE II, categorizing criteria into qualitative and quantitative groups and concluding that qualitative factors^13^ play a crucial role in supplier evaluation. The CSFs were prioritized to implement TQM in the pharmaceutical industry^14^, focusing on analysis, management commitment, supplier relationships, and customer focus. The extended fuzzy TOPSIS addresses MCDM problems with unequal criteria weights, specifically applied to a manager selection problem^15^. This method uses IVFS and performs defuzzification to enhance decision-making accuracy. A fuzzy TOPSIS model^16^ using TFNs was developed for pharmaceutical supplier selection, addressing regional requirements and decision-making uncertainty. For hospital pharmacy supplier selection^17^, the FAHP-FTOPSIS model was established to evaluate suppliers based on cost, delivery, service, flexibility, and relationships. By representing linguistic judgments as triangular fuzzy numbers, this model helped pharmacy managers make more efficient decisions. Additionally, the MILP model is incorporated into FTOPSIS and PCA to streamline supplier selection by reducing data dimensionality^18^. A review of MCDM approaches^19^ for green supplier selection highlights the dominance of fuzzy-based models and the frequent use of environmental management systems as a key criterion. A fuzzy MCDM-MOMILP framework was developed^20^ to optimize supplier selection and order allocation in the pharmaceutical industry, emphasizing sustainability and resilience. An advanced BWM-TOPSIS method with Scenario-Varying Z-numbers and reversed PageRank addressed the impact of Dependent Uncertain Events (DUEs) on GRID supplier selection^21^.Hybrid energy systems were evaluated using TOPSIS and VIKOR to determine the most feasible configuration for powering a health center, highlighting MCDM’s effectiveness in renewable energy selection^22^. Another integrated MCDM approach using SERVQUAL, SF-AHP, SF-WASPAS, and Borda-Copeland assessed hospital performance during COVID-19^23^, providing a benchmark for service quality evaluation. Moreover, a MOSO model for supplier selection^24^ accounted for cost and carbon emission uncertainties, optimizing cost minimization and on-time delivery while highlighting the role of demand forecasting. DEA-ANFIS-PSO and DEA-ANFIS-GA approaches were introduced for pharmaceutical supplier evaluation, as DEA-BCC measured efficiency, and ANFIS predicted supplier performance. The ANFIS-PSO model demonstrated superior accuracy^25^, aiding supplier evaluations and contract renewals. A hybrid fuzzy MADM model combined BWM and ARAS for pharmaceutical supplier selection, focusing on LARG criteria and COVID-19 disruptions^26^, revealing shifts in supplier priorities during different stages of the crisis. An MCDM-SVR-GP model was designed to minimize reliance on expert judgment and enhance supplier performance assessment^27^. A study applied the TOPSIS method to assess suppliers’ green performance in the pharmaceutical industry^28^, identifying leading eco-friendly suppliers. A flexible TOPSIS-GRA approach under an SVN environment was applied for green supplier selection in manufacturing^29^, enhancing decision adaptability in dynamic environments. Various fuzzy decision-making models and optimization methods have been developed for pharmaceutical supplier selection, focusing on managing qualitative and quantitative criteria under uncertainty. However, further integration of methodologies is needed to address emerging challenges in pharmaceuticals. Expanding the selection criteria and enhancing decision-making accuracy are crucial. Additionally, advanced fuzzy numbers are required to more effectively capture subjective judgments and accurately represent supplier performances. Addressing these research gaps, the proposed work offers significant contributions to improving pharmaceutical supplier selection.

### Motivation and contribution

Despite extensive research on supplier selection, it remains a complex decision-making process due to the inherent uncertainty and interdependence of criteria. Previous studies have predominantly focused on sustainability and resilience in supplier evaluation^20^, yet essential aspects such as specialized operational capabilities and efficient stock management remain largely unexplored. Pharmaceutical supplier selection is further complicated by various sources of fuzziness, including the subjective assessment of qualitative criteria, inconsistent data, interdependent factors, uncertainty in supplier performance, and external environmental factors. To address these challenges, advanced uncertainty-handling techniques are required. IVTFS offers a more refined approach to managing uncertainty^5^, yet their application in pharmaceutical supplier selection remains limited. Additionally, integrating machine learning techniques within MCDM frameworks has significantly improved decision-making accuracy and precision^27^. For instance, MARCOS and tree-based machine learning have been used for supplier selection^30^, enabling automated weight calculation in an oil and gas case study. Motivated by these gaps, this study introduces an innovative methodology for enhancing supplier selection in the pharmaceutical sector. The key contributions of this work are outlined below:

i. Leveraged IVTFS to minimize fuzziness in supplier evaluation, enhancing accuracy in pharmaceutical supplier selection.
ii. Expanded selection criteria by incorporating specialized operational factors such as technical expertise, innovation, and inventory management, alongside sustainability and resilience.
iii. Implemented a defuzzification approach for IVTFN, ensuring greater transparency and consistency in decision-making.
iv. Applied SVR to determine criteria weights within the FTOPSIS-GRA framework, improving the precision and objectivity of weight assignment.
v. Collected and analyzed real-time pharmacy data to validate the model, offering practical insights into supplier selection.
vi. Developed a scalable and adaptable framework by integrating fuzzy logic, machine learning, and MCDM techniques to effectively address the complexities of supplier selection in the dynamic pharmaceutical sector.

## Theoretical and methodological advancements

The application of the CFCS algorithm for IVFNs remains unexplored and is advanced in this study. While FTOPSIS and GRA have been widely applied for ranking alternatives, their combined effectiveness in a fuzzy environment has received limited attention in supplier selection. Additionally, most supplier selection studies focus on supply chains and manufacturing^29^, with limited emphasis on pharmaceutical-specific factors. This research addresses these gaps by integrating FTOPSIS-GRA, where FTOPSIS determines proximity to an ideal solution, and GRA ensures relational ranking consistency among alternatives. This methodological combination under an interval-valued fuzzy environment enhances decision robustness by capturing ambiguity through interval-based membership values, making it particularly relevant for pharmaceutical supplier selection. Incorporating pharmaceutical inventory-based criteria introduces a novel and essential dimension to supplier selection, as it captures industry-specific constraints and regulatory requirements.

### Significance of IVTFN

Experts often struggle to precisely quantify their opinions within the interval [0, 1] in fuzzy set theory. To overcome this challenge, Sambuc^31^ and Grattan^32^ emphasized the need for a more comprehensive representation beyond standard fuzzy sets. Gorzlczany^5^ and Turksen^33^ introduced IVFS, later refined by Wang and Li^34^ through the development of IVFN and their extended operations. Conventional fuzzy numbers rely on a single membership function, which may limit their ability to represent uncertainty. IVFNs address this limitation by representing expert opinions as intervals, providing a more comprehensive expression of uncertainty. This enhances flexibility, reduces information loss, and improves decision-making reliability. Fuzzy numbers are leveraged across numerous disciplines, extensively in the decision-making process to handle uncertainty and imprecision of the data. The primary distinction between IVTFN and existing fuzzy numbers lies in their structure and the kind of parameters used to describe them. A Triangular fuzzy number relies on three parameters, and a Trapezoidal fuzzy number uses four. However, both approaches provide a single shape for modeling uncertainty. IVTFN enhances this by employing an interval-based structure, representing the parameter’s upper and lower bounds. This interval approach captures a broader range of possible values and reflects varying degrees. IVTFN offers greater flexibility, enhancing the precision and reliability of decision-making in complex MCDM scenarios.

### Novelties

This research encompasses the following novel aspects list,

i. The concept innovates by merging TOPSIS-GRA in an IVTF environment.
ii. The integrated TOPSIS-GRA methods are incorporated with the SVR and the novel model of SVR-FTOPSIS-GRA is utilized to rank the preferred alternatives.
iii. The defuzzification method for IVTFNs is derived
iv. This work introduces pioneering criteria such as technical expertise, innovation, availability, and inventory tracking capabilities, with the existing of criteria economic viability, environmental impact, and social responsibility. The relevant sub-criteria are incorporated into the supplier performance assessment.

The structure of this work is as follows: The introduction outlines the research background. The literature review summarizes related works and identifies research gaps. The preliminaries of IVFS are then detailed to provide foundational concepts. This is followed by the algorithm of a proposed FTOPSIS-SVR-GRA method. The supplier selection criteria and data availability are then described. The application of the proposed method is illustrated, and the results are presented and discussed in results and discussion. Finally, concludes the work with key findings and suggestions for future research.

## Preliminaries

### Definition 1

**Interval-valued fuzzy set(IVFS)**^5^ Let $X=\{x_1,x_2,\ldots x_n\}$ be a universe of discourse. An interval-valued fuzzy set (IVFS) in *X* is an expression T given by $T=\{x_i,f_A(x_i)\mid x_i \in X\}$, where $f_A:X\rightarrow [0,1]$. The interval $f_A (x_i)$ represents the degree of membership of the element $x_i$ in the set *T*, such that $x_i \rightarrow f_T(x_i )=[f_{A}^{L}(x_i),f_A^U(x_i)]$ where $0\le f_A^L(x_i)\le f_A^U\le 1$. The set of all interval-valued fuzzy set on *X* is denoted by IVFS(*X*). An interval-valued fuzzy set ${\tilde{A}}$ on a universe *X* is defined as: ${\tilde{A}}=\{x,[\mu _{{\tilde{T}}^L} (x_i),\mu _{{\tilde{A}}^U} (x_i)],\mid x_i\in X\}$ where $\mu _{{\tilde{A}}^L}(x_i)$ and $\mu _{{\tilde{A}}^U}(x_i)$ represents the lower bounds and upper bounds of the membership degree for each $x_i\in X$, and satisfy $0\le \mu _{{\tilde{A}}^L} (x_i) \le \mu _{{\tilde{A}}^U} (x_i) \le 1$. Thus, $[\mu _{{\tilde{A}}^L}(x_i),\mu _{{\tilde{A}}^U}(x_i)]$ is an interval within[0,1], representing the membership degree of the element $x_i$ in the set ${\tilde{A}}$.

**Figure 2:**
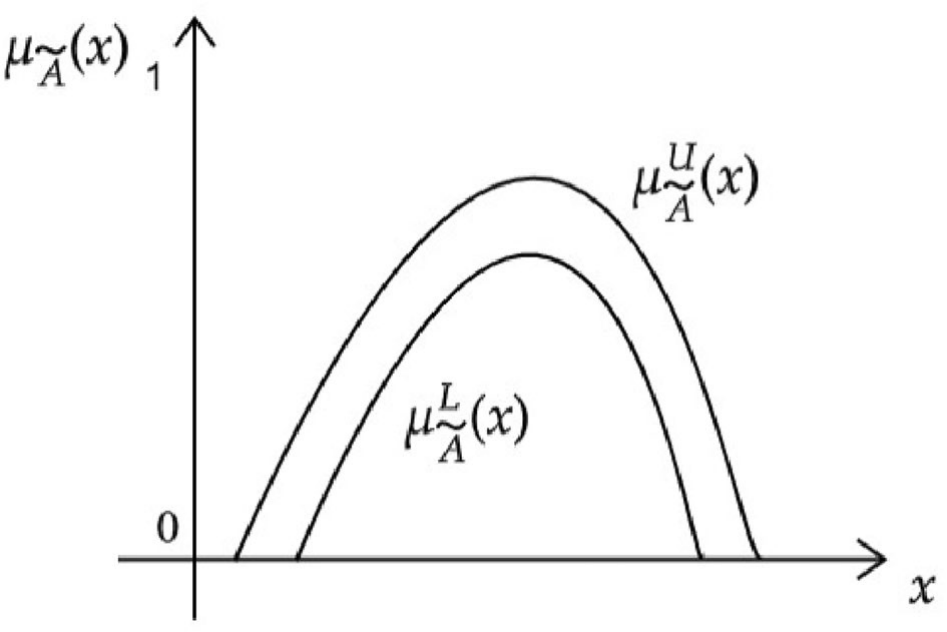
Interval-valued fuzzy set ${\tilde{T}}$.

### Definition 2

**Interval-valued triangular fuzzy number (IVTFN)** ^35^

**Figure 3:**
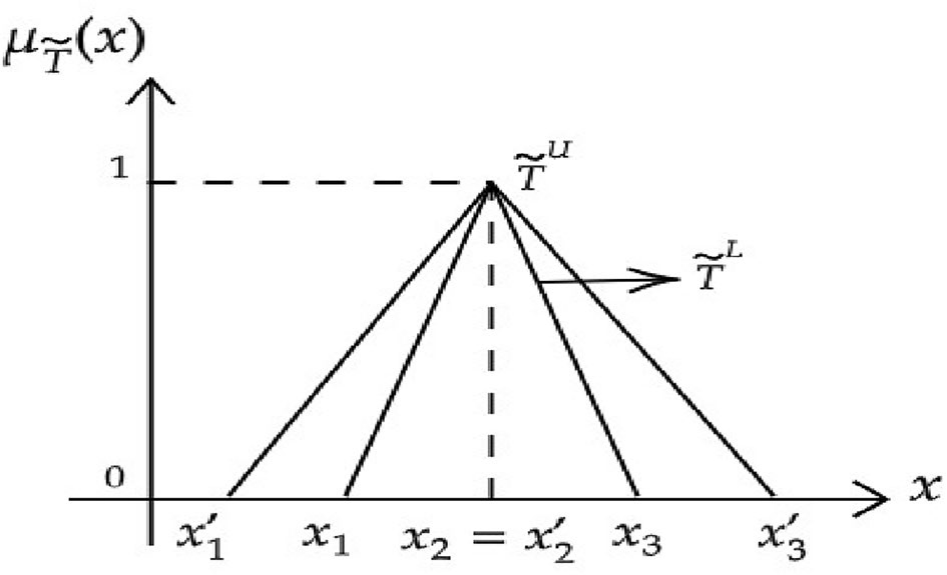
An interval-valued triangular fuzzy number.

Interval-valued triangular fuzzy number can be represented in the set ${\tilde{T}}$, ${\tilde{T}}=[{\tilde{T}}_x^L,{\tilde{T}}_x^U]=[(x_1,x_2,x_3;w_{{\tilde{T}}}),(x_{1}^{\prime },x_{2}^{\prime },x_{3}^{\prime };w^{\prime }_{{\tilde{T}}})]$ as shown in Fig. 2. Where, ${\tilde{T}}^L$ denotes the lower interval-valued triangular fuzzy numbers and ${\tilde{T}}^U$ denotes the upper interval-valued triangular fuzzy numbers. The relations can be obtained as follows.

i. If lower IVTFNs equals upper interval-valued triangular fuzzy numbers ${\tilde{T}}^L={\tilde{T}}^U$, then the IVTFNs ${\tilde{T}}$ is a generalized triangular fuzzy number.
ii. If the interval membership ${x_{1}^{\prime }}=x_1={x_{2}^{\prime }}=x_2={x_{3}^{\prime }}=x_3$ and ${w_{T}^{\prime }}={w_{T}}$ then the IVTFNs ${\tilde{T}}$ is a crisp value.
iii. If ${w_{T}^{\prime }}={w_{T}}=1$ and the core interval $x_2={x_{2}^{\prime }}$, then it denotes the IVTFN ${\tilde{T}}$ as ${\tilde{T}}=[{\tilde{T}}_x^L,{\tilde{T}}_x^U]=[(x_1,x_{1}^{\prime }),(x_{2}^{\prime }=x_2),(x_{3}^{\prime },x_3)]$ the IVTFN can be represented as ${\tilde{T}}=[(x_1,x_{1}^{\prime } ),x_2,(x_{3}^{\prime },x_3)]$. The graphical representation of IVTF are shown in Figs. 2 and 3

## An integrated interval-valued triangular fuzzy TOPSIS-SVR-GRA algorithm

**Figure 4:**
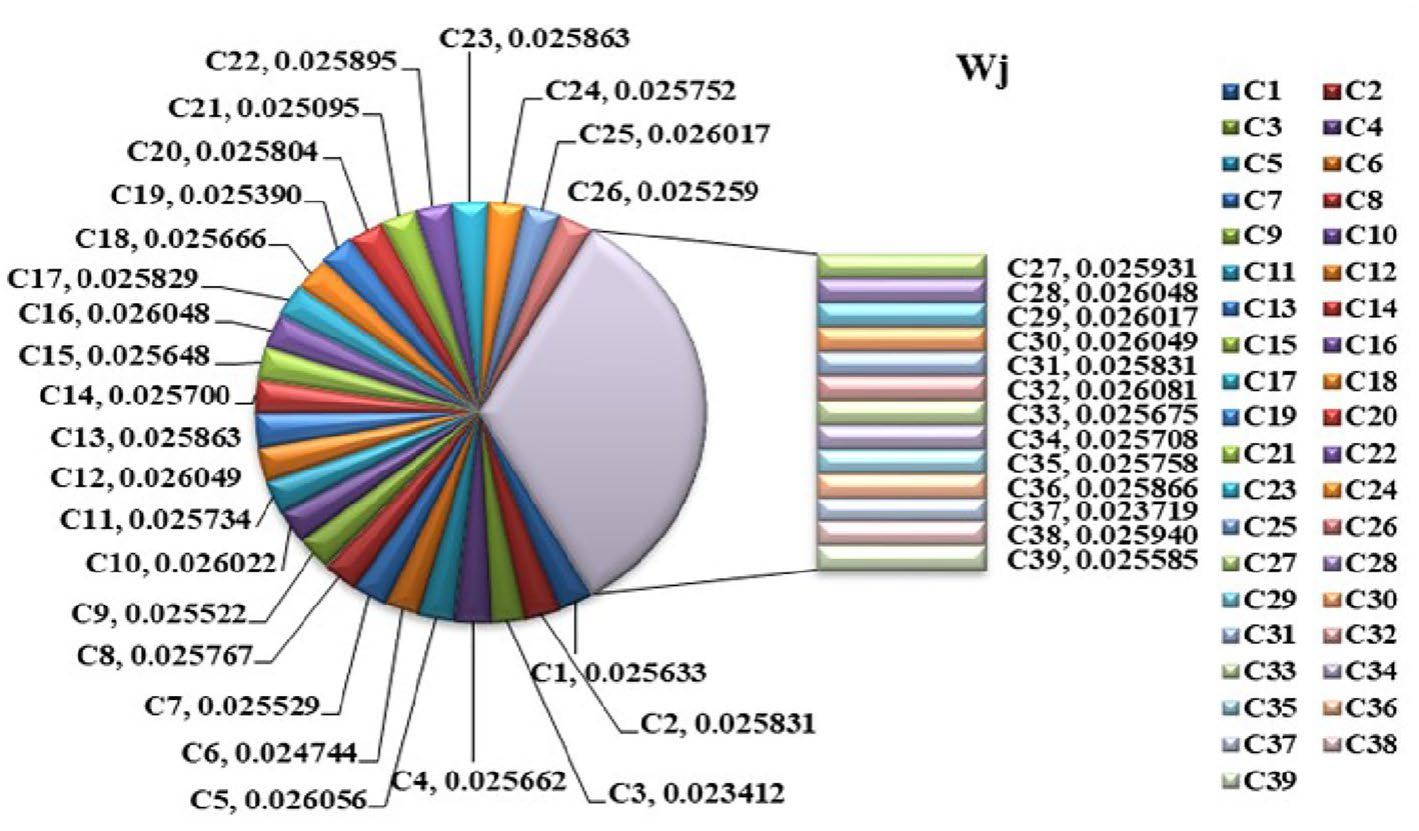
Weighted criteria values.

Step 1: Determine the criteria and alternatives focusing on the sustainability and performance of pharmacy suppliers. Establish the decision-makers for the evaluation process. Let $S_i=\{S_1,S_2,\ldots S_m\}$ be the set of alternatives and $C_j=\{C_1,C_2,\ldots C_n\}$ be the set of criteria and the decision-makers are $D_k=\{D_1,D_2,\ldots D_d\}$
Step 2: Frame the linguistic decision Matrices $L_k$ based on evaluations from experts.
  $$\begin{aligned} L_k= \begin{bmatrix} {\tilde{S}}_{11_{k}} & {\tilde{S}}_{12_{k}} & \dots & {\tilde{S}}_{1n_{k}} \\ {\tilde{S}}_{21_{k}} & {\tilde{S}}_{22_{k}} & \dots & {\tilde{S}}_{2n_{k}} \\ {\tilde{S}}_{m1_{k}} & {\tilde{S}}_{m2_{k}} & \dots & {\tilde{S}}_{mn_{k}} \end{bmatrix} \end{aligned}$$
  Where, $${\tilde{S}}_{ij_{k}}, i=1,2,\dots m,j=1,2,\dots n,k=1,2,\dots d$$
Step 3: Convert Linguistic Matrices into IVTFNs and construct the Interval-Valued Triangular Decision Matrix
  $$\begin{aligned} T= \begin{bmatrix} (x_{1_{11}},x_{1_{11}}^{\prime }),x_{2_{11}},(x_{3_{11}}^{\prime },x_{3_{11}}) & (x_{1_{12}},x_{1_{12}}^{\prime }),x_{2_{12}},(x_{3_{12}}^{\prime },x_{3_{12}}) & \tiny \dots & (x_{1_{1n}},x_{1_{1n}}^{\prime }),x_{2_{1n}},(x_{3_{1n}}^{\prime },x_{3_{1n}})\\ (x_{1_{21}},x_{1_{21}}^{\prime }),x_{2_{21}},(x_{3_{21}}^{\prime },x_{3_{21}}) & (x_{1_{22}},x_{1_{22}}^{\prime }),x_{2_{22}},(x_{3_{22}}^{\prime },x_{3_{22}}) & \tiny \dots & (x_{1_{2n}},x_{1_{2n}}^{\prime }),x_{2_{2n}},(x_{3_{2n}}^{\prime },x_{3_{2n}})\\ \vdots & \vdots & & \vdots \\ (x_{1_{m1}},x_{1_{m1}}^{\prime }),x_{2_{m1}},(x_{3_{m1}}^{\prime },x_{3_{m1}}) & (x_{1_{m2}},x_{1_{m2}}^{\prime }),x_{2_{m2}},(x_{3_{m2}}^{\prime },x_{3_{m2}}) & \tiny \dots & (x_{1_{mn}},x_{1_{mn}}^{\prime }),x_{2_{mn}},(x_{3_{mn}}^{\prime },x_{3_{mn}})\\ \end{bmatrix} \end{aligned}$$
  The evaluation scale is outlined in Tables 1, 2, and 3.
Step 4: The Interval-Valued Triangular Fuzzy matrix is defuzzified into a crisp value matrix using the proposed CFCS algorithm.
  - Normalization of IVTF matrix $({\tilde{T}}_{ij})^n=[(a_{ij}^{\prime } )^{n},(a_{ij})^{n}]$ and $x=[x^{\prime },x]$
    $$\begin{aligned} x a_{ij}^n= & \frac{a_{ij}^n - \min a_{ij}^n}{\Delta _{\min }^{\max }} \quad \text {and} \quad x'(a_{ij}')^n = \frac{(a_{ij}')^n - \min (a_{ij}')^n}{\Delta _{\min }^{\max }} \\ x b_{ij}^n= & \frac{b_{ij}^n - \min b_{ij}^n}{\Delta _{\min }^{\max }} \quad \text {and} \quad x'(b_{ij}')^n = \frac{(b_{ij}')^n - \min (b_{ij}')^n}{\Delta _{\min }^{\max }} \\ x c_{ij}^n= & \frac{c_{ij}^n - \min c_{ij}^n}{\Delta _{\min }^{\max }} \quad \text {and} \quad x'(c_{ij}')^n = \frac{(c_{ij}')^n - \min (c_{ij}')^n}{\Delta _{\min }^{\max }} \end{aligned}$$
  - Evaluation of right score and left score
    $$\begin{aligned} x as_{ij}^n= & \frac{xb_{ij}^n}{1+xb_{ij}^n+xa_{ij}^n} \quad \text {and} \quad x'a'(s'_{ij})^n = \frac{x'(b'_{ij})^n}{1+x'(b'_{ij})^n+x'(a'_{ij})^n} \\ x cs_{ij}^n= & \frac{xc_{ij}^n}{1+xc_{ij}^n+xb_{ij}^n} \quad \text {and} \quad x'c'(s'_{ij})^n = \frac{x'(c'_{ij})^n}{1+x'(c'_{ij})^n+x'(b'_{ij})^n} \end{aligned}$$
  - Calculation of the final normalized values
    $$\begin{aligned} x_{ij}^n=\frac{x as_{ij}^n(1-x as_{ij}^n)+(x cs_{ij}^n)^2}{1-x as_{ij}^n+x cs_{ij}^n} \quad \text {and} \quad (x'_{ij})^n=\frac{xa(s'_{ij})^n(1-xa(s'_{ij})^n)+(xc(s'_{ij})^n)^2}{1-x as_{ij}^n+x cs_{ij}^n} \end{aligned}$$
  - Computation of separated values
    $$\begin{aligned} Z_{ij}^n= & \min a_{ij}^n+x_{ij}^n \text {*} \Delta _{min}^{max} \quad \text {and} \quad (Z'_{ij})^n=\min (a'_{ij})^n+(x'_{ij})^n \text {*} \Delta _{min}^{max}\\ {\tilde{Z}}_{ij}^n= & \frac{Z_{ij}^n + (Z'_{ij})^n}{2} = {\tilde{s}}_{ij} \end{aligned}$$
Step 5: Normalize the defuzzified decision Matrix
  $$\begin{aligned} & \begin{bmatrix} s_{11_{k}} & s_{12_{k}} & \dots & s_{1n_{k}} \\ s_{21_{k}} & s_{22_{k}} & \dots & s_{2n_{k}} \\ \vdots & \vdots & & \vdots \\ s_{m1_{k}} & s_{m2_{k}} & \dots & s_{mn_{k}} \end{bmatrix} = \begin{bmatrix} {\tilde{s}}_{11_{k}} & {\tilde{s}}_{12_{k}} & \dots & {\tilde{s}}_{1n_{k}} \\ {\tilde{s}}_{21_{k}} & {\tilde{s}}_{22_{k}} & \dots & {\tilde{s}}_{2n_{k}} \\ \vdots & \vdots & & \vdots \\ {\tilde{s}}_{m1_{k}} & {\tilde{s}}_{m2_{k}} & \dots & {\tilde{s}}_{mn_{k}} \end{bmatrix} \\ & s_{ij}=\frac{{\tilde{s}}_{ij}}{\sqrt{\sum _{j=1}^{n} {\tilde{s}}_{ij}^2}} i=1,2,3 \dots m\\ & N= \begin{bmatrix} s_{11} & s_{12} & \dots & s_{1n} \\ s_{21} & s_{22} & \dots & s_{2n} \\ \vdots & \vdots & & \vdots \\ s_{m1} & s_{m2} & \dots & s_{mn} \end{bmatrix} \end{aligned}$$
Step 6: Construct the weighted normalized decision matrix using weights predicted by the Support Vector Regressor
  Stage 1: Import necessary libraries
  import pandas as pd from sklearn.preprocessing import StandardScaler from sklearn.model_selection import train_test_split from sklearn.svm import SVR
  Stage 2: Load the dataset
  The dataset is read from a CSV file into a pandas DataFrame df = pd.read_csv(’data_file.csv’)
  Stage 3: Clean column names
  Strip any leading or trailing spaces from column names to ensure consistency in naming df.columns = df.columns.str.strip()
  Stage 4: Extract features and target variable
  Select the feature columns (S1-S7) and the target column (weights) from the dataset X = df[[’S1’, ’S2’, ’S3’, ’S4’, ’S5’, ’S6’,.. ’Sn’]] y = df[’weights’] Target variable is ’weights’
  Stage 5: Normalize the feature data
  Use StandardScaler to normalize the feature columns (S1-Sn) to have a mean of 0 and standard deviation of 1 scaler = StandardScaler() X_scaled = scaler.fit_transform(X)
  Stage 6: Split data into training and testing sets
  Split the normalized data into training (80 X_train, X_test, y_train, y_test = train_test_split(X_scaled, y, test_size=0.2, random_state=42)
  Stage 7: Initialize and train the SVR model
  Create an SVR model with an RBF kernel, and train it using the training data svr = SVR(kernel=’rbf’, C=1.0, epsilon=0.1) svr.fit(X_train, y_train)
  Stage 8: Make predictions on the entire dataset
  Use the trained SVR model to predict the weights for all records in the dataset df[’Predicted_Weight’] = svr.predict(X_scaled)
  Stage 9: Save the predicted results Save the updated dataset with the predicted weights to a new CSV file df.to_csv(’predicted_results.csv’, index=False)
  $$\begin{aligned} w_j= & \{w_1,w_2,\dots w_n \} \\ W= & \begin{bmatrix} w_1s_{11} & w_2s_{12} & \dots & w_ns_{1n} \\ w_1s_{21} & w_2s_{22} & \dots & w_ns_{2n} \\ \vdots & \vdots & & \vdots \\ w_1s_{m1} & w_2s_{m2} & \dots & w_ns_{mn} \end{bmatrix} =\begin{bmatrix} Z_{11} & Z_{12} & \dots & Z_{1n} \\ Z_{21} & Z_{22} & \dots & Z_{2n} \\ \vdots & \vdots & & \vdots \\ Z_{m1} & Z_{m2} & \dots & Z_{mn} \end{bmatrix} \end{aligned}$$
  $\text {where},Z_{ij}=s_{ij}\times w_{ij}, i=1,2,\dots ,m  \text {and} j=1,2,\dots n$ The criteria weights are visually represented as pie charts in Figs. 4, 8 and 10 and in the figures $\{C_1, C_2,..C_{39}\}$ represents the determined 39 criteria.The input data (X) consists of supplier performance scores across multiple criteria, while the output data (Y) represents expert-defined criteria weights. SVR is trained on the supplier dataset to learn the relationship between supplier performance and the importance assigned to each criterion, ensuring a data-driven and consistent weight-determination process
Step 7: Determine Fuzzy positive ideal solution (FPIS) and Fuzzy negative ideal solution (FNIS)
  $$\begin{aligned} A^+= & \{ z_1^+, \dots , z_n^+ \} = \{ (\max _j \, s_{ij} \mid i \in I'), (\min _j \, s_{ij} \mid i \in I'') \} \\ A^-= & \{ z_1^-, \dots , z_n^- \} = \{ (\max _j \, s_{ij} \mid i \in I'), (\min _j \, s_{ij} \mid i \in I'') \} \end{aligned}$$
  $I^{\prime }$ denotes Benefit attributes
  $I^{''}$ denotes Cost attributes
Step 8: Calculate the Grey relational co-efficient
  $$\begin{aligned} r(z^+(j), z_i(j)) = \frac{\min _i \min _j |z^+(j) - z_i(j)| + \zeta \max _i \max _j |z^+(j) - z_i(j)|}{z^+(j) - z_i(j) + \zeta \max _i \max _j |z^+(j) - z_i(j)|} \end{aligned}$$
  $$\begin{aligned} r(z^-(j), z_i(j)) = \frac{\min _i \min _j |z^-(j) - z_i(j)| + \zeta \max _i \max _j |z^-(j) - z_i(j)|}{z^-(j) - z_i(j) + \zeta \max _i \max _j |z^-(j) - z_i(j)|} \end{aligned}$$
Step 9: Calculate the closeness co-efficient
  $$\begin{aligned} r_i^+= & r(z^+, z_i) = \sum _{j=1}^n \omega _j r(z^+(j), z_i(j)) \\ r_i^-= & r(z^-, z_i) = \sum _{j=1}^n \omega _j r(z^-(j), z_i(j)), \quad \sum _{j=1}^n \omega _j = 1 \end{aligned}$$
Step 10: Rank the alternatives.
  $$\begin{aligned} C_i = \frac{r_i^+}{r_i^+ + r_i^-} \end{aligned}$$

**Table 1:** Linguistic variables in terms of IVTF for $P_1$.

Linguistic terms	IVTFN
Very Low(*NS*)	[(0,0.05);0.15;(0.25,0.3)]
Low(*BA*)	[(0.20,0.25);0.35;(0.45,0.5)]
Medium(*A*)	[(0.40,0.45);0.55;(0.65,0.70)]
High(*AA*)	[(0.60,0.65);0.75;(0.85,0.90)]
Very High(*S*)	[(0.80,0.85);0.90;(0.95,1)]

**Table 2:** Linguistic evaluation scale in terms of IVTF for $P_2$.

Linguistic terms	IVTFN
*NS*	[(0,0.025);0.10;(0.165,0.20)]
*BA*	[(0.05,0.125);0.30;(0.365,0.40)]
*A*	[(0.25,0.325);0.50;(0.565,0.60)
*AA*	[(0.45,0.525);0.7;(0.765,0.80)]
*S*	[(0.65,0.725);0.9;(0.965,1)]

**Table 3:** Linguistic scale with corresponding IVTF for $P_3$.

Linguistic terms	IVTFN
*NS*	[(0,0.025);0.10;(0.2,0.25)]
*BA*	[(0.05,0.15);0.3;(0.4,0.45)]
*A*	[(0.25,0.35);0.5;(0.6,0.65)]
*AA*	[(0.45,0.55);0.7;(0.8,0.85)]
*S*	[(0.65,0.75);0.9;(0.95,1)]

**Figure 5:**
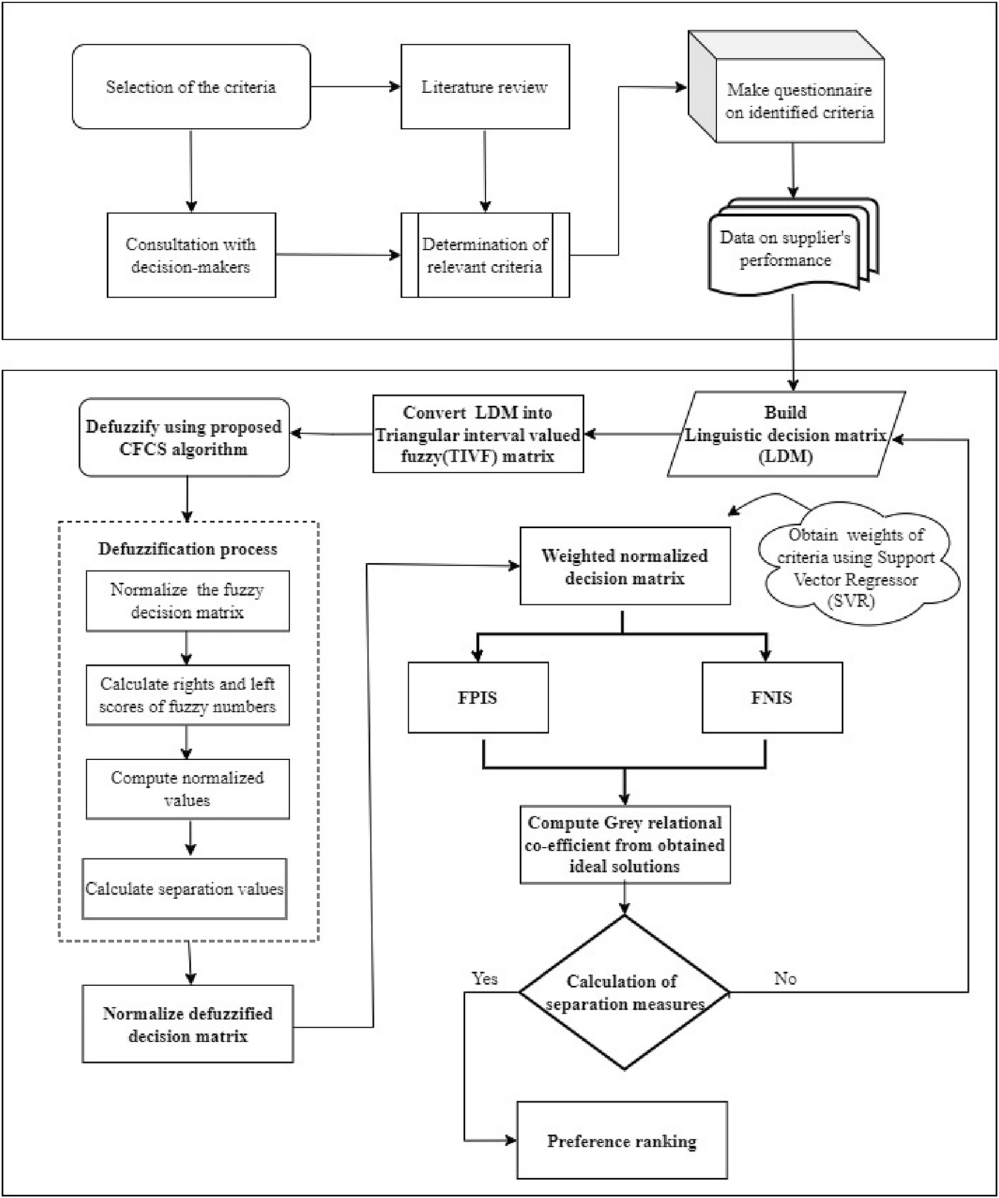
Architecture of the proposed approach.

## Implementation of an integrated approach for the selection of sustainable suppliers in pharmaceuticals

In the scope of pharmaceutical supplier selection, developing an efficient, sustainable, and holistic evaluation framework is crucial. The proposed framework enhances the decision-making process by incorporating a systematic approach that aligns with the operational needs and strategic goals of pharmaceutical stores. This section explains the critical supplier criteria and the data collection process.

### Determination of supplier evaluation criteria

Appropriate criteria selection is crucial as it forms the foundation for effective supplier evaluation. A thorough examination of previous studies^13,16,18^ on pharmaceutical supplier selection^14,20,25,28^ helped to identify key performance indicators. Moreover, the experts’ feedback ensured that the chosen criteria aligned with real-world procurement challenges. In this work, criteria are incorporated based on key dimensions including economic viability, environmental impact, social responsibility, product responsibility, availability, ability assessment, technical expertise, innovation, and inventory tracking capabilities. By prioritizing sub-criteria within these dimensions, a structured approach is established to compare and select the best suppliers (alternatives) among the competitors. The relevant criteria and its representations include, Cost structure$(C_{1})$, Credit period$(C_{2})$,Cash back offers$(C_{3})$, Delivery$(C_{4})$, Service$(C_{5})$, Discounts$(C_{6})$, Carbon footprint$(C_{7})$, Energy Efficiency$(C_{8})$, Community Engagement$(C_{9})$, Good Distribution Practice(GDP)$(C_{10})$, Brand reputation and Trust$(C_{11})$, Certification$(C_{12})$, Continuous quality improvement$(C_{13})$, Regulatory Compliance$(C_{14})$, Product Quality$(C_{15})$, Product Safety$(C_{16})$, Product effectiveness$(C_{17})$, Defect rates$(C_{18})$, Product wise discount$(C_{19})$, Long life product$(C_{20})$, Fixed-dose combination$(C_{21})$, Good Bioavailability product$(C_{22})$, Financial stability$(C_{23})$, Track record$(C_{24})$, Supplier Quality Assurance$(C_{25})$, Immediate supply$(C_{26})$, Corrective action$(C_{27})$, Experience$(C_{28})$, Responsiveness$(C_{29})$, Adequate Qualification$(C_{30})$, Strategic Collaboration$(C_{31})$, Quality Control Systems$(C_{32})$, Versatility$(C_{33})$, Tech Trend Awareness$(C_{34})$, Research and Development (R&D) implication$(C_{35})$, Combo medication procurement$(C_{36})$, JIT delivery$(C_{37})$, Lead time$(C_{38})$ and Storage space$(C_{39})$. Figure 7 shows the hierarchical structure representing the goal, criteria, sub-criteria, and the alternatives. This approach is based on the fact that the significance of criteria can vary both across different groups and within the same group. Assigning weights at the group level could lead to an oversimplified representation, where criteria within a group might be assumed to have similar importance. Instead, assigning weights at the individual level ensures that each criterion is evaluated based on its actual impact rather than being influenced by its category. Although the grouping does not directly affect the weight computation, it is utilized as a framework for structuring and interpreting the evaluation process.The mentioned criteria are considered according to the expert’s (decision-makers in pharmaceutical stores) consultations and through the comprehensive literature review.

### Data collection

Supplier’s performance data were gathered by establishing pioneering criteria from three reputable retail pharmaceutical stores, with a sample size of $n=33 (7+11+15)$ suppliers. The criteria include economic viability, environmental impact, social and product responsibility, availability, capability assessment, technical expertise, innovation, and inventory management. The linguistic rating scale was employed based on IVTFNs presented in Tables 1, 2, and 3. This fuzzy scale enables a flexible representation of pharmaceutical expert opinions. Experts’ insights on supplier performance are captured through five linguistic terms, namely Not Satisfactory (NS), Below Average (BA), Average (A), Above Average (AA), and Satisfactory (S). The linguistic terms^36^ were adopted to reflect the expert’s subjective judgments. This approach ensures that the collected primary data represents each supplier’s performance in fulfilling the operational requirements of pharmaceuticals. The sourced supplier linguistic performances are constructed as an LDM in Tables 4, 7, 10, and 11. The overall representation of the proposed model and the hierarchical structure are shown in Figs. 5 and 7.

## Supplier selection

To demonstrate the implementation of the proposed framework, the selection process of suppliers is illustrated employing data collected from three pharmaceutical stores and labeled $P_{1}$,$P_{2}$, and $P_{3}$. Supplier evaluation is based on the key criteria considered. The alternatives represent individuals or entities supplying the requirements of a pharmaceutical store. In the linguistic decision matrix (LDM) tables supplier performances are categorized as satisfactory (S) for very high, above average(AA) for high, average (A)for medium, below average (BA) for low, and not satisfactory (NS) for very low. The performance of each supplier is evaluated based on LDM and transformed into a fuzzy decision matrix for more accurate decision-making.

### Numerical illustration of $P_{1}$

**Figure 6:**
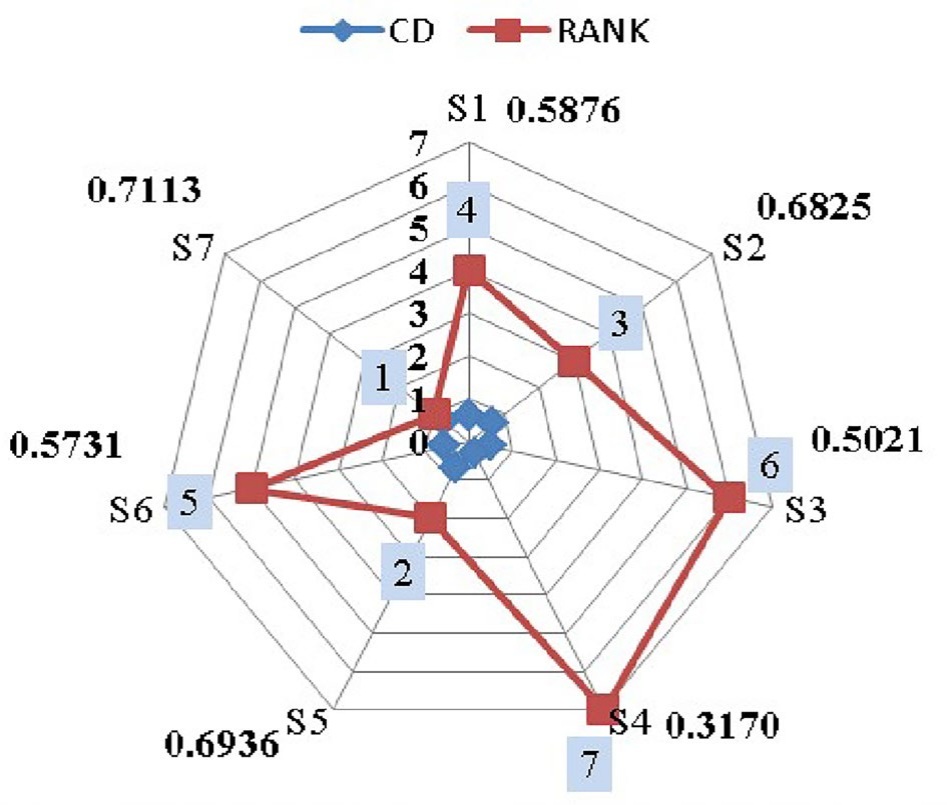
Ranking of alternatives.

Step 1 : Frame the alternative and criteria. The alternatives are the seven suppliers in the pharmaceutical store $(P_{1})$. Let $S_{i}=\{S_{1},S_{2},\ldots S_{7}\}$ be the set of alternatives and $C_{j}=\{C_{1},C_{2},\ldots C_{39}\}$ be the set of criteria and the decision-makers are $D_k=\{D_{1},D_{2}\}$.
Step 2 : Transform the collected performance data into an LDM in Table 4.
Step 3 : Convert the LDM into interval-valued triangular fuzzy matrix. Then, construct the aggregated IVTF decision matrix
Step 4 : Convert interval-valued triangular fuzzy values into crisp values by utilizing the CFCS algorithm. The defuzzification process involves normalizing the IVTF matrix, evaluating right and left scores to quantify fuzzy numbers, calculating final normalized values, and computing separated values for accurate and effective analysis. The resulting defuzzified matrix is presented in Supplementary Table 1.
Step 5 : Normalization of the defuzzified matrix is obtained and given in Supplementary Table 2.
Step 6 : Determine the weighted decision matrix using SVR and obtained in Supplementary Table 3.
Step 7 : Calculated PIS and NIS of the alternatives and shown in Table 5
Step 8 : Estimate the relative coefficient using GRA.
Step 9 : Determine the closeness co-efficient degree CD and obtain the ranking of the suppliers in the preference order shown in Table 6 and represented in Fig. 6.

**Table 4:** Linguistic decision matrix.

	$D_1$							$D_2$						
	$S_1$	$S_2$	$S_3$	$S_4$	$S_5$	$S_6$	$S_7$	$S_1$	$S_2$	$S_3$	$S_4$	$S_5$	$S_6$	$S_7$
$C_1$	*A*	*AA*	*A*	*A*	*AA*	*AA*	*AA*	*A*	*AA*	*A*	*A*	*S*	*S*	*BA*
$C_2$	*AA*	*AA*	*AA*	*AA*	*A*	*A*	*A*	*AA*	*A*	*S*	*AA*	*A*	*AA*	*A*
$C_3$	*NS*	*A*	*NS*	*BA*	*AA*	*AA*	*AA*	*NS*	*A*	*NS*	*A*	*AA*	*AA*	*AA*
$C_4$	*A*	*S*	*AA*	*AA*	*S*	*S*	*S*	*BA*	*AA*	*AA*	*AA*	*S*	*AA*	*S*
$C_5$	*AA*	*AA*	*S*	*S*	*S*	*S*	*S*	*AA*	*S*	*AA*	*AA*	*S*	*AA*	*S*
$C_6$	*BA*	*A*	*AA*	*NS*	*AA*	*AA*	*AA*	*NS*	*A*	*A*	*BA*	*A*	*A*	*A*
$C_7$	*AA*	*A*	*A*	*AA*	*A*	*A*	*A*	*A*	*AA*	*AA*	*A*	*S*	*BA*	*NS*
$C_8$	*A*	*A*	*BA*	*A*	*A*	*A*	*A*	*A*	*A*	*A*	*A*	*S*	*A*	*S*
$C_9$	*A*	*AA*	*S*	*BA*	*AA*	*AA*	*AA*	*AA*	*AA*	*AA*	*BA*	*AA*	*AA*	*A*
$C_{10}$	*AA*	*S*	*AA*	*S*	*S*	*S*	*S*	*AA*	*S*	*AA*	*AA*	*S*	*S*	*S*
$C_{11}$	*S*	*S*	*AA*	*A*	*S*	*S*	*S*	*S*	*S*	*A*	*A*	*S*	*S*	*S*
$C_{12}$	*AA*	*AA*	*AA*	*AA*	*S*	*S*	*S*	*AA*	*S*	*S*	*S*	*S*	*S*	*S*
$C_{13}$	*AA*	*AA*	*AA*	*A*	*AA*	*AA*	*AA*	*A*	*S*	*S*	*A*	*S*	*S*	*AA*
$C_{14}$	*A*	*AA*	*S*	*A*	*A*	*A*	*A*	*A*	*AA*	*AA*	*A*	*AA*	*A*	*A*
$C_{15}$	*AA*	*S*	*S*	*BA*	*S*	*S*	*S*	*AA*	*S*	*AA*	*A*	*S*	*AA*	*S*
$C_{16}$	*S*	*S*	*AA*	*AA*	*S*	*S*	*S*	*S*	*S*	*S*	*AA*	*S*	*AA*	*S*
$C_{17}$	*A*	*AA*	*A*	*A*	*AA*	*AA*	*AA*	*A*	*S*	*AA*	*A*	*AA*	*AA*	*AA*
$C_{18}$	*BA*	*A*	*A*	*A*	*AA*	*AA*	*AA*	*BA*	*A*	*A*	*BA*	*A*	*A*	*BA*
$C_{19}$	*BA*	*A*	*A*	*NS*	*S*	*S*	*S*	*A*	*A*	*A*	*NS*	*A*	*A*	*A*
$C_{20}$	*AA*	*AA*	*AA*	*BA*	*AA*	*AA*	*AA*	*AA*	*AA*	*A*	*A*	*AA*	*AA*	*AA*
$C_{21}$	*BA*	*S*	*BA*	*BA*	*AA*	*AA*	*AA*	*A*	*S*	*A*	*A*	*S*	*AA*	*S*
$C_{22}$	*AA*	*AA*	*AA*	*AA*	*S*	*S*	*S*	*AA*	*AA*	*A*	*A*	*AA*	*S*	*S*
$C_{23}$	*A*	*A*	*S*	*AA*	*AA*	*AA*	*AA*	*A*	*AA*	*AA*	*AA*	*S*	*S*	*S*
$C_{24}$	*A*	*AA*	*A*	*A*	*S*	*S*	*S*	*A*	*AA*	*AA*	*A*	*AA*	*AA*	*AA*
$C_{25}$	*A*	*AA*	*AA*	*AA*	*AA*	*AA*	*AA*	*AA*	*S*	*S*	*AA*	*S*	*AA*	*S*
$C_{26}$	*BA*	*A*	*A*	*BA*	*S*	*S*	*S*	*A*	*AA*	*AA*	*A*	*AA*	*S*	*S*
$C_{27}$	*AA*	*AA*	*AA*	*A*	*A*	*A*	*A*	*S*	*AA*	*AA*	*A*	*S*	*AA*	*S*
$C_{28}$	*AA*	*S*	*AA*	*AA*	*S*	*S*	*S*	*S*	*S*	*S*	*AA*	*S*	*S*	*S*
$C_{29}$	*AA*	*AA*	*AA*	*A*	*AA*	*AA*	*AA*	*AA*	*S*	*AA*	*AA*	*S*	*S*	*S*
$C_{30}$	*S*	*S*	*AA*	*AA*	*S*	*S*	*S*	*S*	*S*	*S*	*AA*	*AA*	*AA*	*S*
$C_{31}$	*A*	*AA*	*A*	*A*	*AA*	*AA*	*AA*	*AA*	*AA*	*A*	*A*	*S*	*AA*	*A*
$C_{32}$	*AA*	*AA*	*S*	*S*	*S*	*S*	*S*	*S*	*S*	*S*	*S*	*S*	*S*	*S*
$C_{33}$	*A*	*AA*	*AA*	*BA*	*AA*	*AA*	*AA*	*A*	*AA*	*A*	*A*	*AA*	*S*	*AA*
$C_{34}$	*BA*	*AA*	*A*	*A*	*A*	*A*	*A*	*A*	*S*	*A*	*AA*	*S*	*AA*	*S*
$C_{35}$	*BA*	*A*	*A*	*A*	*AA*	*AA*	*AA*	*A*	*S*	*AA*	*AA*	*S*	*AA*	*AA*
$C_{36}$	*AA*	*AA*	*A*	*AA*	*S*	*S*	*S*	*AA*	*S*	*A*	*AA*	*S*	*AA*	*AA*
$C_{37}$	*NS*	*AA*	*BA*	*BA*	*S*	*S*	*S*	*NS*	*AA*	*BA*	*BA*	*AA*	*AA*	*AA*
$C_{38}$	*BA*	*A*	*A*	*A*	*AA*	*AA*	*AA*	*S*	*AA*	*AA*	*A*	*AA*	*BA*	*BA*
$C_{39}$	*A*	*AA*	*A*	*A*	*S*	*S*	*S*	*A*	*AA*	*A*	*AA*	*S*	*S*	*S*

**Table 5:** PIS and NIS value.

Criteria	$r^+$	$r^-$	Criteria	$r^+$	$r^-$
$C_1$	0.0080	0.0119	$C_{21}$	0.0124	0.0062
$C_2$	0.0118	0.0079	$C_{22}$	0.0113	0.0081
$C_3$	0.0117	0.0023	$C_{23}$	0.0107	0.0071
$C_4$	0.0111	0.0056	$C_{24}$	0.0111	0.0074
$C_5$	0.0106	0.0088	$C_{25}$	0.0104	0.0082
$C_6$	0.0111	0.0043	$C_{26}$	0.0121	0.0060
$C_7$	0.0056	0.0116	$C_{27}$	0.0113	0.0075
$C_8$	0.0119	0.0074	$C_{28}$	0.0103	0.0086
$C_9$	0.0115	0.0049	$C_{29}$	0.0104	0.0082
$C_{10}$	0.0104	0.0087	$C_{30}$	0.0104	0.0087
$C_{11}$	0.0106	0.0065	$C_{31}$	0.0118	0.0079
$C_{12}$	0.0104	0.0087	$C_{32}$	0.0101	0.0092
$C_{13}$	0.0107	0.0071	$C_{33}$	0.0117	0.0064
$C_{14}$	0.0128	0.0085	$C_{34}$	0.0121	0.0066
$C_{15}$	0.0108	0.0054	$C_{35}$	0.0116	0.0063
$C_{16}$	0.0103	0.0086	$C_{36}$	0.0113	0.0069
$C_{17}$	0.0116	0.0077	$C_{37}$	0.0115	0.0021
$C_{18}$	0.0062	0.0116	$C_{38}$	0.0087	0.0118
$C_{19}$	0.0116	0.0056	$C_{39}$	0.0115	0.0070
$C_{20}$	0.0104	0.0063			

**Figure 7:**
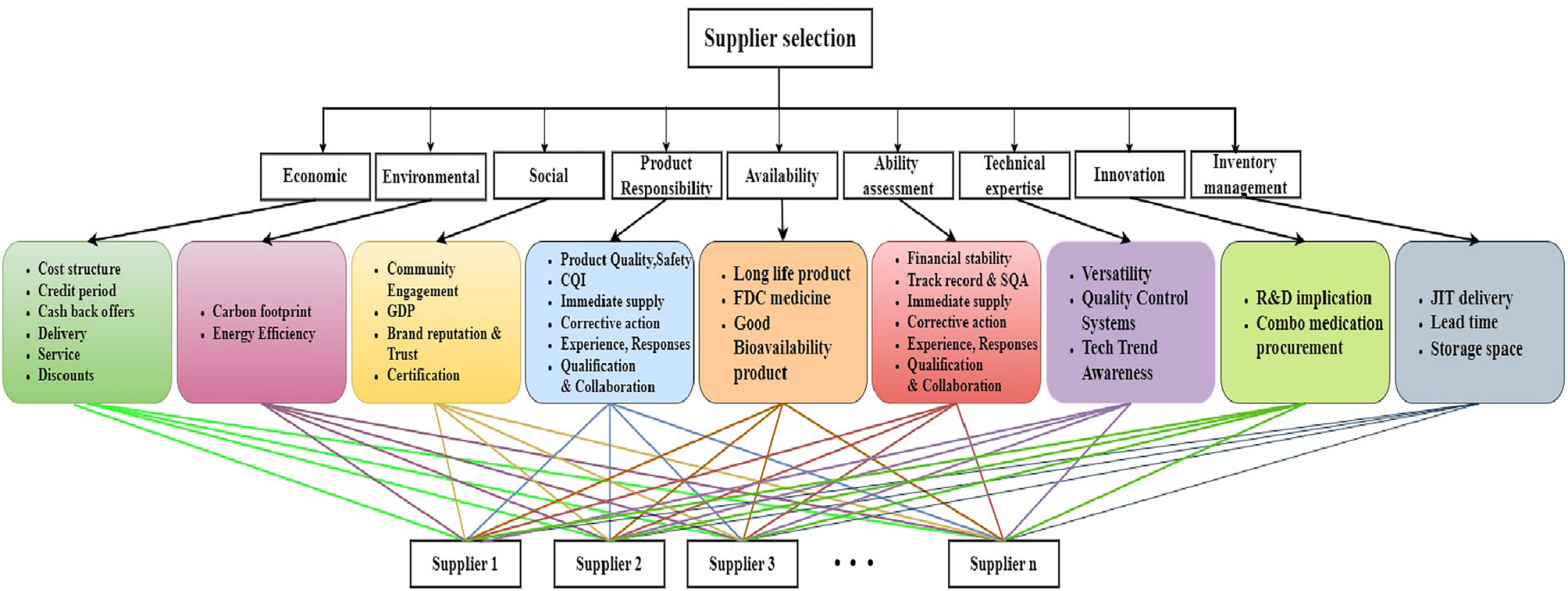
Hierarchical structure.

**Table 6:** Integrated closeness coefficient and ranking.

**Suppliers**	$w_j^* r_i^+$	$w_j^* r_i^-$	**CD**	**RANK**
$S_1$	1.5126	1.0616	0.5876	4
$S_2$	1.7082	0.7948	0.6825	3
$S_3$	1.0972	1.0881	0.5021	6
$S_4$	0.8107	1.7470	0.3170	7
$S_5$	1.8207	0.8043	0.6936	2
$S_6$	1.2767	0.9511	0.5731	5
$S_7$	1.8431	0.7479	0.7113	1

### Numerical illustration of $P_{2}$

Step 1: Frame the alternative and criteria. The alternatives are the eleven suppliers for the pharmaceutical store $(P_{2})$. Let $S_{i}=\{S_{1},S_{2},\ldots S_{11}\}$ be the set of alternatives and $C_{j}=\{C_{1},C_{2},\ldots C_{39}\}$ be the set of criteria and the decision-makers are $D_k=\{D_{1},D_{2}\}$
Step 2: Convert the gathered performance data into a LDM in the Table 7
Step 3: Transform the LDM into IVTF matrix. Then build the aggregated IVTF decision matrix
Step 4: Utilizing CFCS algorithm to convert IVTF values into precise values and generate the defuzzified matrix in Supplementary Table 4.
Step 5: Normalized the defuzzified matrix and represented in the Supplementary Table 5.
Step 6: Compute the weighted decision matrix using SVR in the Supplementary Table 6.
Step 7: Calculate PIS and NIS and represent in the Table 9.
Step 8: Estimate the relative coefficient using GRA.
Step 9: Calculate the closeness co-efficient CD and obtain the ranking of the suppliers in the preference order shown in Table 8 and represented in Fig. 9.

**Table 7:** Suppliers Performance on expert’s opinion.

	$D_{1}$											$D_{2}$										
	$S_1$	$S_2$	$S_3$	$S_4$	$S_5$	$S_6$	$S_7$	$S_8$	$S_9$	$S_{10}$	$S_{11}$	$S_1$	$S_2$	$S_3$	$S_4$	$S_5$	$S_6$	$S_7$	$S_8$	$S_9$	$S_{10}$	$S_{11}$
$C_1$	*A*	*A*	*A*	*A*	*AA*	*BA*	*A*	*BA*	*BA*	*BA*	*BA*	*A*	*BA*	*AA*	*AA*	*A*	*AA*	*BA*	*NS*	*AA*	*AA*	*A*
$C_2$	*BA*	*A*	*A*	*AA*	*AA*	*A*	*A*	*A*	*BA*	*A*	*BA*	*AA*	*A*	*AA*	*AA*	*A*	*A*	*A*	*BA*	*BA*	*A*	*BA*
$C_3$	*BA*	*BA*	*A*	*AA*	*A*	*BA*	*A*	*A*	*BA*	*A*	*A*	*AA*	*NS*	*AA*	*AA*	*BA*	*NS*	*A*	*NS*	*BA*	*AA*	*A*
$C_4$	*A*	*BA*	*A*	*A*	*AA*	*A*	*A*	*BA*	*BA*	*A*	*A*	*A*	*BA*	*AA*	*A*	*A*	*AA*	*AA*	*A*	*A*	*AA*	*BA*
$C_5$	*A*	*BA*	*AA*	*AA*	*AA*	*BA*	*BA*	*A*	*A*	*A*	*A*	*AA*	*A*	*AA*	*AA*	*BA*	*A*	*A*	*AA*	*A*	*AA*	*BA*
$C_6$	*BA*	*NS*	*AA*	*AA*	*AA*	*BA*	*BA*	*A*	*A*	*BA*	*A*	*AA*	*A*	*AA*	*S*	*A*	*NS*	*A*	*AA*	*BA*	*A*	*A*
$C_7$	*A*	*NS*	*A*	*A*	*A*	*BA*	*BA*	*A*	*BA*	*BA*	*BA*	*AA*	*A*	*A*	*A*	*BA*	*AA*	*AA*	*A*	*AA*	*AA*	*BA*
$C_8$	*A*	*BA*	*A*	*A*	*A*	*BA*	*A*	*BA*	*A*	*BA*	*BA*	*A*	*BA*	*A*	*A*	*A*	*A*	*BA*	*BA*	*A*	*AA*	*A*
$C_9$	*A*	*NS*	*AA*	*AA*	*AA*	*BA*	*BA*	*A*	*A*	*BA*	*BA*	*AA*	*BA*	*A*	*A*	*NS*	*BA*	*A*	*A*	*BA*	*A*	*AA*
$C_{10}$	*A*	*BA*	*AA*	*A*	*AA*	*A*	*A*	*A*	*A*	*A*	*A*	*AA*	*A*	*AA*	*AA*	*A*	*A*	*AA*	*A*	*A*	*A*	*BA*
$C_{11}$	*A*	*BA*	*AA*	*A*	*A*	*A*	*A*	*A*	*BA*	*A*	*BA*	*AA*	*A*	*AA*	*AA*	*BA*	*A*	*AA*	*BA*	*AA*	*A*	*A*
$C_{12}$	*A*	*BA*	*AA*	*A*	*A*	*A*	*BA*	*BA*	*BA*	*A*	*BA*	*AA*	*AA*	*A*	*AA*	*A*	*A*	*A*	*AA*	*A*	*AA*	*A*
$C_{13}$	*A*	*NS*	*AA*	*A*	*AA*	*A*	*A*	*A*	*BA*	*BA*	*A*	*A*	*BA*	*AA*	*A*	*A*	*A*	*A*	*A*	*A*	*AA*	*A*
$C_{14}$	*A*	*NS*	*A*	*A*	*A*	*A*	*A*	*A*	*BA*	*BA*	*A*	*A*	*BA*	*A*	*BA*	*BA*	*BA*	*A*	*BA*	*A*	*BA*	*A*
$C_{15}$	*A*	*BA*	*A*	*A*	*A*	*A*	*A*	*A*	*A*	*BA*	*BA*	*AA*	*A*	*AA*	*AA*	*A*	*AA*	*AA*	*A*	*A*	*A*	*A*
$C_{16}$	*A*	*BA*	*A*	*A*	*A*	*BA*	*A*	*BA*	*BA*	*BA*	*A*	*A*	*A*	*AA*	*A*	*A*	*AA*	*A*	*A*	*A*	*AA*	*A*
$C_{17}$	*A*	*BA*	*A*	*A*	*A*	*A*	*A*	*BA*	*A*	*BA*	*A*	*A*	*BA*	*A*	*A*	*A*	*A*	*A*	*BA*	*A*	*A*	*BA*
$C_{18}$	*A*	*BA*	*A*	*AA*	*A*	*A*	*BA*	*BA*	*BA*	*A*	*BA*	*A*	*NS*	*A*	*A*	*BA*	*BA*	*AA*	*BA*	*A*	*A*	*A*
$C_{19}$	*A*	*NS*	*AA*	*AA*	*AA*	*BA*	*A*	*A*	*BA*	*A*	*BA*	*AA*	*NS*	*AA*	*S*	*A*	*NS*	*BA*	*NS*	*AA*	*A*	*A*
$C_{20}$	*AA*	*NS*	*AA*	*AA*	*A*	*BA*	*AA*	*BA*	*BA*	*BA*	*BA*	*A*	*A*	*A*	*AA*	*BA*	*AA*	*BA*	*BA*	*A*	*A*	*BA*
$C_{21}$	*A*	*BA*	*A*	*A*	*AA*	*BA*	*BA*	*A*	*BA*	*A*	*BA*	*AA*	*A*	*A*	*A*	*A*	*A*	*A*	*AA*	*BA*	*A*	*A*
$C_{22}$	*A*	*A*	*AA*	*A*	*AA*	*A*	*AA*	*A*	*BA*	*BA*	*A*	*AA*	*A*	*AA*	*AA*	*A*	*S*	*AA*	*A*	*A*	*A*	*BA*
$C_{23}$	*S*	*A*	*AA*	*AA*	*AA*	*A*	*AA*	*A*	*BA*	*A*	*BA*	*AA*	*A*	*AA*	*AA*	*AA*	*A*	*AA*	*BA*	*A*	*BA*	*A*
$C_{24}$	*S*	*BA*	*A*	*A*	*A*	*BA*	*A*	*BA*	*BA*	*BA*	*BA*	*AA*	*A*	*A*	*A*	*A*	*A*	*A*	*A*	*A*	*A*	*A*
$C_{25}$	*AA*	*NS*	*A*	*A*	*A*	*BA*	*BA*	*BA*	*BA*	*BA*	*BA*	*AA*	*A*	*A*	*A*	*A*	*A*	*A*	*A*	*A*	*A*	*BA*
$C_{26}$	*AA*	*NS*	*A*	*A*	*AA*	*BA*	*BA*	*A*	*A*	*BA*	*BA*	*A*	*BA*	*A*	*A*	*A*	*AA*	*A*	*A*	*A*	*A*	*BA*
$C_{27}$	*AA*	*NS*	*A*	*A*	*A*	*BA*	*BA*	*A*	*BA*	*BA*	*A*	*A*	*BA*	*AA*	*AA*	*BA*	*A*	*A*	*A*	*A*	*A*	*A*
$C_{28}$	*A*	*NS*	*AA*	*A*	*AA*	*A*	*BA*	*BA*	*BA*	*BA*	*A*	*A*	*AA*	*AA*	*AA*	*BA*	*BA*	*BA*	*A*	*BA*	*AA*	*BA*
$C_{29}$	*A*	*NS*	*AA*	*AA*	*A*	*BA*	*A*	*A*	*BA*	*BA*	*BA*	*AA*	*BA*	*AA*	*A*	*BA*	*BA*	*A*	*BA*	*A*	*BA*	*BA*
$C_{30}$	*BA*	*NS*	*AA*	*A*	*AA*	*BA*	*BA*	*A*	*A*	*A*	*A*	*AA*	*A*	*AA*	*BA*	*A*	*BA*	*BA*	*A*	*BA*	*A*	*A*
$C_{31}$	*A*	*BA*	*AA*	*A*	*A*	*A*	*A*	*BA*	*BA*	*BA*	*A*	*AA*	*BA*	*AA*	*BA*	*A*	*AA*	*AA*	*A*	*A*	*A*	*BA*
$C_{32}$	*A*	*NS*	*AA*	*A*	*A*	*BA*	*A*	*BA*	*BA*	*BA*	*BA*	*AA*	*A*	*AA*	*BA*	*A*	*A*	*AA*	*A*	*A*	*A*	*BA*
$C_{33}$	*AA*	*A*	*AA*	*A*	*AA*	*A*	*A*	*A*	*BA*	*BA*	*BA*	*AA*	*A*	*A*	*A*	*A*	*A*	*A*	*BA*	*A*	*AA*	*A*
$C_{34}$	*A*	*BA*	*A*	*A*	*AA*	*A*	*A*	*BA*	*BA*	*BA*	*BA*	*A*	*BA*	*AA*	*A*	*A*	*BA*	*AA*	*A*	*A*	*A*	*BA*
$C_{35}$	*A*	*NS*	*AA*	*A*	*A*	*BA*	*A*	*A*	*BA*	*BA*	*A*	*AA*	*A*	*AA*	*A*	*BA*	*AA*	*BA*	*A*	*BA*	*A*	*A*
$C_{36}$	*A*	*NS*	*A*	*AA*	*AA*	*A*	*A*	*BA*	*BA*	*BA*	*BA*	*AA*	*A*	*AA*	*AA*	*A*	*BA*	*A*	*A*	*A*	*A*	*A*
$C_{37}$	*A*	*NS*	*A*	*AA*	*AA*	*BA*	*BA*	*A*	*BA*	*BA*	*A*	*AA*	*NS*	*AA*	*S*	*BA*	*NS*	*BA*	*NS*	*AA*	*BA*	*A*
$C_{38}$	*AA*	*NS*	*A*	*AA*	*AA*	*A*	*A*	*A*	*A*	*A*	*BA*	*A*	*A*	*AA*	*A*	*A*	*AA*	*AA*	*BA*	*BA*	*BA*	*BA*
$C_{39}$	*A*	*NS*	*AA*	*AA*	*AA*	*BA*	*BA*	*BA*	*BA*	*BA*	*A*	*AA*	*BA*	*A*	*BA*	*A*	*A*	*A*	*BA*	*A*	*AA*	*A*

**Table 8:** Closeness index and suppliers ranking.

**Suppliers**	$w_j^* r_i^+$	$w_j^* r_i^-$	**CD**	**Rank**
$S_1$	0.7620	0.4138	0.6481	2
$S_2$	0.4314	0.7526	0.3644	11
$S_3$	0.7756	0.4158	0.6510	1
$S_4$	0.7274	0.4350	0.6258	3
$S_5$	0.6514	0.4553	0.5886	4
$S_6$	0.4864	0.6657	0.4222	9
$S_7$	0.6544	0.4919	0.5709	5
$S_8$	0.4878	0.6159	0.4420	8
$S_9$	0.4513	0.7041	0.3906	10
$S_{10}$	0.5329	0.5842	0.4770	7
$S_{11}$	0.5539	0.5696	0.4930	6

**Table 9:** PIS and NIS.

**Criteria**	$r^+$	$r^-$	**Criteria**	$r^+$	$r^-$
$C_1$	0.0023	0.0102	$C_{21}$	0.0098	0.0048
$C_2$	0.0108	0.0045	$C_{22}$	0.0111	0.0055
$C_3$	0.0113	0.0032	$C_{23}$	0.0104	0.0038
$C_4$	0.0091	0.0044	$C_{24}$	0.0127	0.0046
$C_5$	0.0099	0.0056	$C_{25}$	0.0123	0.0035
$C_6$	0.0115	0.0029	$C_{26}$	0.0111	0.0018
$C_7$	0.0051	0.0100	$C_{27}$	0.0104	0.0053
$C_8$	0.0089	0.0052	$C_{28}$	0.0104	0.0065
$C_9$	0.0098	0.0033	$C_{29}$	0.0116	0.0033
$C_{10}$	0.0100	0.0056	$C_{30}$	0.0100	0.0048
$C_{11}$	0.0103	0.0043	$C_{31}$	0.0098	0.0033
$C_{12}$	0.0090	0.0059	$C_{32}$	0.0111	0.0046
$C_{13}$	0.0108	0.0031	$C_{33}$	0.0095	0.0039
$C_{14}$	0.0094	0.0038	$C_{34}$	0.0112	0.0038
$C_{15}$	0.0091	0.0060	$C_{35}$	0.0124	0.0042
$C_{16}$	0.0097	0.0064	$C_{36}$	0.0126	0.0042
$C_{17}$	0.0086	0.0050	$C_{37}$	0.0105	0.0035
$C_{18}$	0.0034	0.0119	$C_{38}$	0.0059	0.0106
$C_{19}$	0.0114	0.0016	$C_{39}$	0.0089	0.0044
$C_{20}$	0.0111	0.0032			

**Figure 8:**
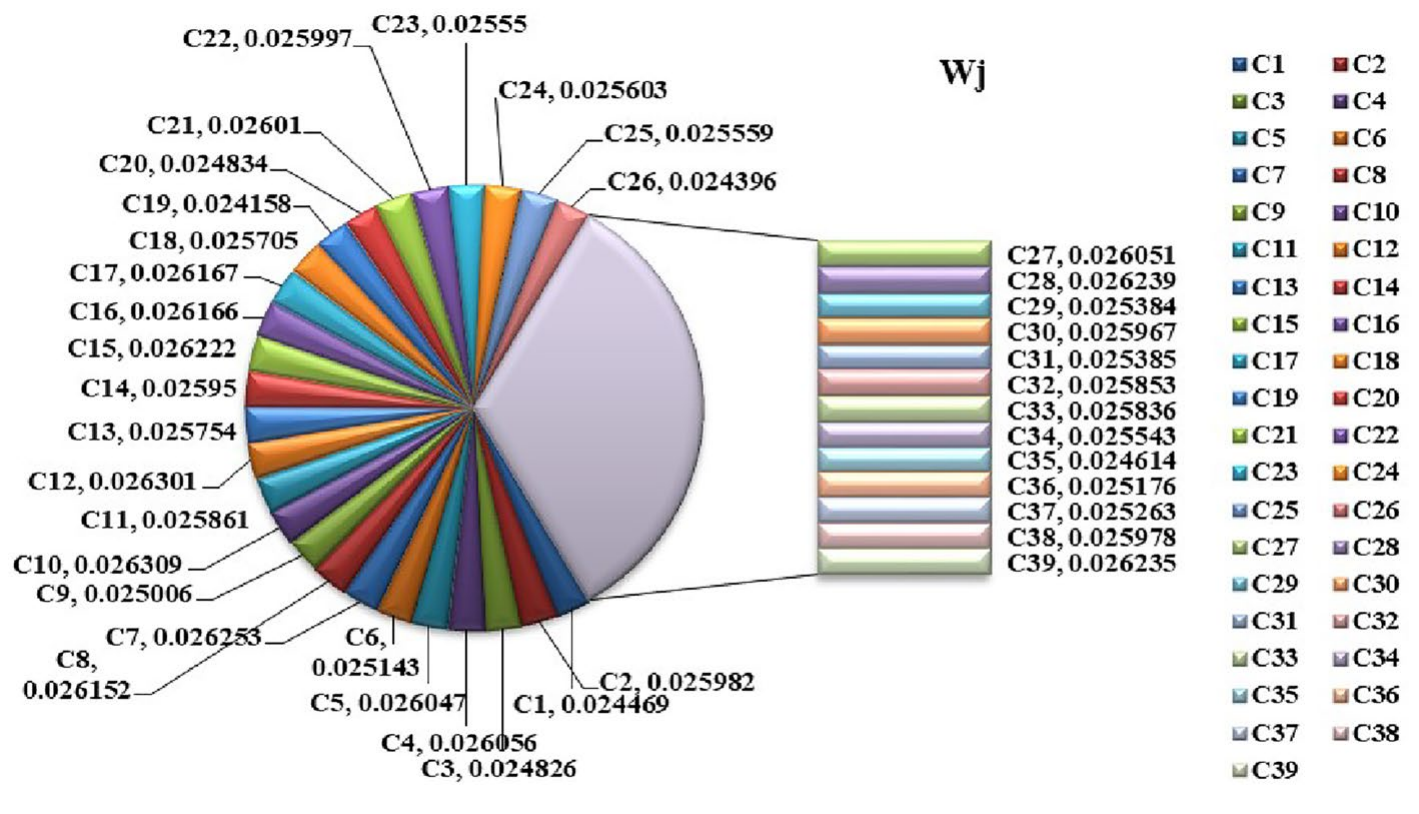
Relative importance of criteria.

**Figure 9:**
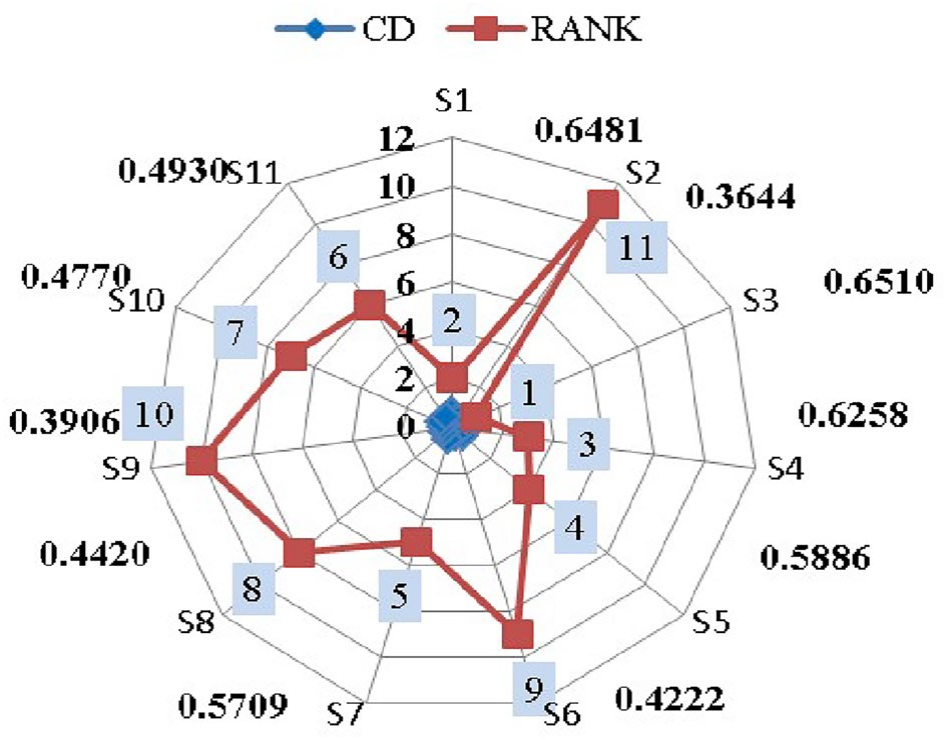
Supplier performance rankings.

### Numerical illustration of $P_{3}$

Step 1 : Frame the alternative and criteria. The alternatives represent fifteen suppliers for the third pharmaceutical store $(P_{3})$. Let $S_i=\{S_{1},S_{2},\ldots S_{15}\}$ be the set of alternatives and $C_j=\{C_1,C_{2},\ldots C_{39}\}$ be the set of criteria and the decision-makers are $D_k=\{D_{1},D_{2}\}$
Step 2 : Transform the collected performance data into an LDM on $D_1$ and $D_2$ opinions’ in Tables 10 and 11.
Step 3 : Convert the LDM into IVTF matrix. Then construct the aggregated IVTF decision matrix.
Step 4 : Convert IVTF values into precise values by utilizing the CFCS algorithm, and the defuzzified matrix is obtained in Supplementary Table 7.
Step 5 : Normalize the defuzzified matrix and represent in Supplementary Table 8.
Step 6 : Determine the weighted decision matrix using SVR in Supplementary Table 9.
Step 7 : Calculate the PIS and NIS and represented in the Table 12
Step 8 : Estimate the relative coefficient using GRA.
Step 9 : Determine the closeness co-efficient and obtain the ranking of suppliers in preference order in Table 13 and represented in Fig. 11

**Table 10:** LDM on decision-makers($D_1$) perspectives.

	$S_1$	$S_2$	$S_3$	$S_4$	$S_5$	$S_6$	$S_7$	$S_8$	$S_9$	$S_{10}$	$S_{11}$	$S_{12}$	$S_{13}$	$S_{14}$	$S_{15}$
$C_1$	*BA*	*A*	*A*	*A*	*A*	*A*	*A*	*A*	*A*	*A*	*A*	*BA*	*A*	*A*	*A*
$C_2$	*A*	*BA*	*BA*	*S*	*BA*	*AA*	*AA*	*BA*	*A*	*AA*	*BA*	*BA*	*A*	*NS*	*BA*
$C_3$	*NS*	*A*	*A*	*A*	*A*	*A*	*NS*	*BA*	*BA*	*A*	*NS*	*NS*	*BA*	*NS*	*BA*
$C_4$	*S*	*NS*	*NS*	*S*	*BA*	*AA*	*AA*	*AA*	*BA*	*AA*	*S*	*A*	*AA*	*AA*	*AA*
$C_5$	*AA*	*NS*	*NS*	*S*	*A*	*AA*	*AA*	*AA*	*A*	*A*	*S*	*A*	*AA*	*AA*	*AA*
$C_6$	*BA*	*BA*	*BA*	*NS*	*BA*	*BA*	*NS*	*NS*	*NS*	*BA*	*NS*	*NS*	*NS*	*BA*	*BA*
$C_7$	*BA*	*BA*	*BA*	*S*	*A*	*A*	*AA*	*AA*	*BA*	*BA*	*A*	*BA*	*NS*	*AA*	*A*
$C_8$	*AA*	*BA*	*BA*	*S*	*A*	*A*	*AA*	*AA*	*BA*	*A*	*AA*	*BA*	*A*	*AA*	*A*
$C_9$	*AA*	*A*	*NS*	*AA*	*A*	*AA*	*S*	*S*	*BA*	*BA*	*S*	*A*	*AA*	*A*	*A*
$C_{10}$	*AA*	*AA*	*A*	*S*	*A*	*AA*	*S*	*S*	*BA*	*A*	*S*	*A*	*AA*	*AA*	*AA*
$C_{11}$	*AA*	*A*	*A*	*A*	*A*	*AA*	*AA*	*AA*	*BA*	*A*	*AA*	*A*	*A*	*AA*	*AA*
$C_{12}$	*AA*	*A*	*A*	*S*	*BA*	*S*	*AA*	*AA*	*BA*	*A*	*AA*	*A*	*AA*	*A*	*AA*
$C_{13}$	*AA*	*AA*	*AA*	*S*	*A*	*S*	*A*	*A*	*BA*	*BA*	*AA*	*A*	*AA*	*AA*	*AA*
$C_{14}$	*AA*	*A*	*A*	*BA*	*NS*	*A*	*A*	*A*	*BA*	*A*	*BA*	*BA*	*BA*	*A*	*AA*
$C_{15}$	*AA*	*AA*	*AA*	*AA*	*A*	*AA*	*AA*	*AA*	*A*	*AA*	*AA*	*A*	*AA*	*A*	*AA*
$C_{16}$	*S*	*AA*	*S*	*AA*	*A*	*AA*	*S*	*S*	*A*	*A*	*S*	*A*	*S*	*AA*	*AA*
$C_{17}$	*AA*	*AA*	*AA*	*A*	*A*	*A*	*A*	*A*	*A*	*A*	*S*	*A*	*S*	*A*	*A*
$C_{18}$	*A*	*A*	*A*	*A*	*A*	*A*	*A*	*A*	*A*	*A*	*A*	*A*	*BA*	*A*	*AA*
$C_{19}$	*AA*	*A*	*S*	*NS*	*A*	*NS*	*A*	*A*	*NS*	*A*	*A*	*NS*	*NS*	*NS*	*BA*
$C_{20}$	*A*	*A*	*A*	*A*	*A*	*A*	*A*	*A*	*A*	*A*	*A*	*A*	*A*	*A*	*A*
$C_{21}$	*A*	*A*	*A*	*A*	*A*	*A*	*A*	*A*	*A*	*A*	*A*	*A*	*A*	*A*	*A*
$C_{22}$	*A*	*A*	*A*	*A*	*A*	*A*	*A*	*A*	*A*	*A*	*A*	*A*	*A*	*A*	*A*
$C_{23}$	*AA*	*AA*	*AA*	*AA*	*BA*	*AA*	*S*	*S*	*AA*	*S*	*A*	*AA*	*AA*	*AA*	*AA*
$C_{24}$	*AA*	*A*	*A*	*S*	*A*	*AA*	*AA*	*S*	*A*	*A*	*AA*	*AA*	*AA*	*AA*	*AA*
$C_{25}$	*AA*	*AA*	*A*	*AA*	*A*	*AA*	*AA*	*AA*	*A*	*AA*	*AA*	*A*	*S*	*AA*	*AA*
$C_{26}$	*A*	*NS*	*NS*	*AA*	*BA*	*AA*	*AA*	*AA*	*BA*	*A*	*S*	*A*	*S*	*AA*	*A*
$C_{27}$	*AA*	*BA*	*A*	*AA*	*BA*	*AA*	*AA*	*A*	*BA*	*A*	*AA*	*A*	*A*	*AA*	*A*
$C_{28}$	*AA*	*S*	*BA*	*S*	*A*	*AA*	*S*	*AA*	*A*	*A*	*AA*	*AA*	*AA*	*AA*	*S*
$C_{29}$	*AA*	*A*	*S*	*A*	*BA*	*S*	*A*	*AA*	*A*	*BA*	*A*	*AA*	*AA*	*A*	*A*
$C_{30}$	*S*	*S*	*A*	*S*	*BA*	*AA*	*S*	*S*	*BA*	*AA*	*S*	*A*	*S*	*S*	*S*
$C_{31}$	*A*	*A*	*AA*	*BA*	*BA*	*AA*	*S*	*S*	*BA*	*A*	*S*	*BA*	*AA*	*AA*	*S*
$C_{32}$	*A*	*BA*	*S*	*A*	*A*	*A*	*A*	*A*	*BA*	*A*	*S*	*A*	*A*	*A*	*A*
$C_{33}$	*AA*	*A*	*A*	*S*	*AA*	*AA*	*S*	*A*	*A*	*AA*	*S*	*A*	*A*	*A*	*A*
$C_{34}$	*AA*	*A*	*S*	*S*	*A*	*S*	*AA*	*AA*	*AA*	*S*	*A*	*S*	*AA*	*A*	*S*
$C_{35}$	*AA*	*S*	*S*	*AA*	*A*	*S*	*S*	*AA*	*BA*	*AA*	*AA*	*S*	*S*	*AA*	*AA*
$C_{36}$	*AA*	*AA*	*AA*	*S*	*BA*	*S*	*A*	*A*	*S*	*A*	*S*	*A*	*S*	*S*	*S*
$C_{37}$	*AA*	*S*	*S*	*S*	*A*	*S*	*S*	*AA*	*AA*	*A*	*S*	*S*	*S*	*S*	*S*
$C_{38}$	*S*	*S*	*S*	*S*	*S*	*S*	*S*	*S*	*S*	*S*	*S*	*S*	*S*	*S*	*S*
$C_{39}$	*S*	*S*	*S*	*S*	*S*	*S*	*S*	*S*	*S*	*S*	*S*	*S*	*S*	*S*	*S*

**Table 11:** LDM on decision-makers($D_2$) perspectives.

	$S_1$	$S_2$	$S_3$	$S_4$	$S_5$	$S_6$	$S_7$	$S_8$	$S_9$	$S_{10}$	$S_{11}$	$S_{12}$	$S_{13}$	$S_{14}$	$S_{15}$
$C_1$	*AA*	*BA*	*AA*	*AA*	*A*	*AA*	*AA*	*A*	*A*	*A*	*AA*	*BA*	*A*	*A*	*A*
$C_2$	*A*	*NS*	*A*	*AA*	*BA*	*A*	*AA*	*A*	*BA*	*A*	*BA*	*BA*	*A*	*A*	*BA*
$C_3$	*AA*	*BA*	*AA*	*BA*	*A*	*BA*	*A*	*A*	*BA*	*A*	*BA*	*A*	*BA*	*BA*	*BA*
$C_4$	*NS*	*A*	*BA*	*AA*	*BA*	*AA*	*AA*	*AA*	*BA*	*AA*	*S*	*S*	*S*	*AA*	*AA*
$C_5$	*AA*	*AA*	*BA*	*BA*	*S*	*BA*	*A*	*AA*	*AA*	*BA*	*AA*	*AA*	*AA*	*A*	*AA*
$C_6$	*NS*	*A*	*BA*	*NS*	*BA*	*NS*	*A*	*NS*	*NS*	*NS*	*BA*	*AA*	*BA*	*BA*	*BA*
$C_7$	*A*	*AA*	*A*	*AA*	*A*	*A*	*A*	*AA*	*A*	*A*	*AA*	*AA*	*BA*	*AA*	*AA*
$C_8$	*S*	*BA*	*BA*	*AA*	*A*	*AA*	*BA*	*AA*	*BA*	*A*	*AA*	*AA*	*A*	*AA*	*AA*
$C_9$	*BA*	*AA*	*BA*	*AA*	*A*	*A*	*A*	*AA*	*A*	*A*	*AA*	*AA*	*S*	*AA*	*AA*
$C_{10}$	*S*	*A*	*A*	*AA*	*A*	*AA*	*AA*	*AA*	*BA*	*A*	*S*	*AA*	*S*	*A*	*AA*
$C_{11}$	*S*	*BA*	*A*	*AA*	*A*	*AA*	*AA*	*AA*	*BA*	*AA*	*S*	*BA*	*S*	*AA*	*AA*
$C_{12}$	*AA*	*AA*	*A*	*AA*	*A*	*A*	*AA*	*AA*	*A*	*AA*	*AA*	*BA*	*S*	*AA*	*AA*
$C_{13}$	*AA*	*AA*	*BA*	*AA*	*A*	*AA*	*A*	*AA*	*BA*	*A*	*A*	*AA*	*S*	*AA*	*AA*
$C_{14}$	*NS*	*AA*	*BA*	*BA*	*S*	*AA*	*A*	*A*	*BA*	*NS*	*BA*	*A*	*A*	*A*	*A*
$C_{15}$	*S*	*AA*	*A*	*AA*	*A*	*A*	*AA*	*AA*	*A*	*A*	*S*	*AA*	*S*	*AA*	*AA*
$C_{16}$	*AA*	*S*	*A*	*AA*	*A*	*A*	*AA*	*BA*	*A*	*AA*	*A*	*A*	*S*	*A*	*S*
$C_{17}$	*S*	*AA*	*A*	*AA*	*A*	*AA*	*A*	*AA*	*A*	*AA*	*AA*	*AA*	*S*	*S*	*AA*
$C_{18}$	*NS*	*BA*	*BA*	*A*	*A*	*A*	*A*	*BA*	*BA*	*A*	*BA*	*BA*	*A*	*A*	*A*
$C_{19}$	*BA*	*AA*	*AA*	*BA*	*NS*	*NS*	*BA*	*A*	*BA*	*A*	*AA*	*BA*	*NS*	*BA*	*BA*
$C_{20}$	*NS*	*AA*	*A*	*A*	*A*	*A*	*A*	*A*	*BA*	*S*	*BA*	*A*	*A*	*A*	*A*
$C_{21}$	*BA*	*AA*	*A*	*A*	*A*	*A*	*A*	*BA*	*A*	*AA*	*AA*	*A*	*A*	*A*	*A*
$C_{22}$	*S*	*BA*	*A*	*A*	*A*	*A*	*A*	*A*	*BA*	*A*	*S*	*AA*	*A*	*A*	*A*
$C_{23}$	*BA*	*AA*	*AA*	*AA*	*A*	*AA*	*S*	*S*	*A*	*S*	*AA*	*A*	*AA*	*A*	*S*
$C_{24}$	*AA*	*A*	*A*	*S*	*A*	*AA*	*AA*	*S*	*A*	*A*	*AA*	*AA*	*AA*	*AA*	*AA*
$C_{25}$	*S*	*AA*	*A*	*S*	*A*	*AA*	*AA*	*AA*	*A*	*A*	*AA*	*AA*	*S*	*AA*	*AA*
$C_{26}$	*A*	*S*	*AA*	*BA*	*A*	*BA*	*A*	*A*	*BA*	*A*	*AA*	*AA*	*S*	*AA*	*A*
$C_{27}$	*AA*	*AA*	*BA*	*AA*	*NS*	*A*	*A*	*A*	*BA*	*A*	*AA*	*AA*	*S*	*A*	*A*
$C_{28}$	*BA*	*AA*	*BA*	*S*	*A*	*A*	*AA*	*AA*	*BA*	*S*	*A*	*A*	*S*	*A*	*A*
$C_{29}$	*S*	*A*	*BA*	*AA*	*A*	*A*	*A*	*AA*	*BA*	*S*	*AA*	*S*	*A*	*A*	*AA*
$C_{30}$	*BA*	*AA*	*BA*	*S*	*A*	*A*	*AA*	*AA*	*BA*	*S*	*AA*	*S*	*A*	*A*	*AA*
$C_{31}$	*BA*	*A*	*A*	*BA*	*A*	*S*	*AA*	*AA*	*BA*	*S*	*AA*	*S*	*AA*	*A*	*S*
$C_{32}$	*S*	*A*	*A*	*S*	*BA*	*AA*	*A*	*S*	*A*	*AA*	*A*	*S*	*A*	*A*	*S*
$C_{33}$	*S*	*AA*	*A*	*A*	*AA*	*A*	*A*	*S*	*AA*	*A*	*S*	*AA*	*AA*	*S*	*A*
$C_{34}$	*A*	*S*	*S*	*S*	*S*	*AA*	*S*	*A*	*AA*	*S*	*S*	*A*	*A*	*A*	*S*
$C_{35}$	*S*	*AA*	*S*	*AA*	*S*	*A*	*S*	*AA*	*S*	*S*	*S*	*AA*	*AA*	*S*	*S*
$C_{36}$	*BA*	*AA*	*A*	*AA*	*AA*	*A*	*S*	*AA*	*S*	*S*	*AA*	*AA*	*S*	*S*	*S*
$C_{37}$	*S*	*S*	*AA*	*S*	*AA*	*A*	*S*	*S*	*S*	*S*	*S*	*S*	*S*	*S*	*S*
$C_{38}$	*S*	*S*	*S*	*S*	*S*	*S*	*S*	*S*	*S*	*S*	*S*	*S*	*S*	*S*	*S*
$C_{39}$	*S*	*S*	*S*	*S*	*S*	*S*	*S*	*S*	*S*	*S*	*S*	*S*	*S*	*S*	*S*

**Table 12:** PIS and NIS values.

Criteria	$r^+$	$r^-$	Criteria	$r^+$	$r^-$
$C_1$	0.00135	0.00281	$C_{21}$	0.00922	0.00600
$C_2$	0.02117	0.00508	$C_{22}$	0.01550	0.00858
$C_3$	0.02039	0.00660	$C_{23}$	0.01802	0.00769
$C_4$	0.00613	0.00130	$C_{24}$	0.00863	0.00467
$C_5$	0.00138	0.00029	$C_{25}$	0.00784	0.00424
$C_6$	0.00532	0.00136	$C_{26}$	0.00204	0.00043
$C_7$	0.00061	0.00255	$C_{27}$	0.00102	0.00028
$C_8$	0.00531	0.00187	$C_{28}$	0.01010	0.00315
$C_9$	0.00161	0.00057	$C_{29}$	0.01021	0.00377
$C_{10}$	0.00348	0.00109	$C_{30}$	0.00428	0.00262
$C_{11}$	0.01585	0.00559	$C_{31}$	0.01764	0.00714
$C_{12}$	0.00977	0.00471	$C_{32}$	0.01813	0.00982
$C_{13}$	0.00299	0.00105	$C_{33}$	0.00486	0.00208
$C_{14}$	0.01906	0.00617	$C_{34}$	0.00625	0.00302
$C_{15}$	0.00585	0.00358	$C_{35}$	0.01615	0.00387
$C_{16}$	0.01805	0.00770	$C_{36}$	0.01202	0.00651
$C_{17}$	0.00574	0.00311	$C_{37}$	0.00325	0.00138
$C_{18}$	0.00322	0.00645	$C_{38}$	0.01866	0.00756
$C_{19}$	0.01448	0.00185	$C_{39}$	0.00381	0.00163
$C_{20}$	0.00384	0.00163

**Table 13:** Alternatives’ closeness coefficient and rankings.

Suppliers	$w_j \cdot r_{i}^{+}$	$w_j \cdot r_{i}^{-}$	CD	RANK
$S_1$	0.6626	0.4850	0.5773	2
$S_2$	0.5515	0.5517	0.4999	10
$S_3$	0.5010	0.6261	0.4445	12
$S_4$	0.6270	0.5345	0.5398	5
$S_5$	0.4315	0.6877	0.3855	14
$S_6$	0.5461	0.5252	0.5097	8
$S_7$	0.6016	0.4915	0.5504	4
$S_8$	0.5887	0.5163	0.5327	6
$S_9$	0.3981	0.7796	0.3380	15
$S_{10}$	0.4525	0.6552	0.4085	13
$S_{11}$	0.6502	0.5100	0.5604	3
$S_{12}$	0.5465	0.5428	0.5017	9
$S_{13}$	0.6867	0.4854	0.5859	1
$S_{14}$	0.5104	0.5706	0.4722	11
$S_{15}$	0.5742	0.5148	0.5273	7

**Figure 10:**
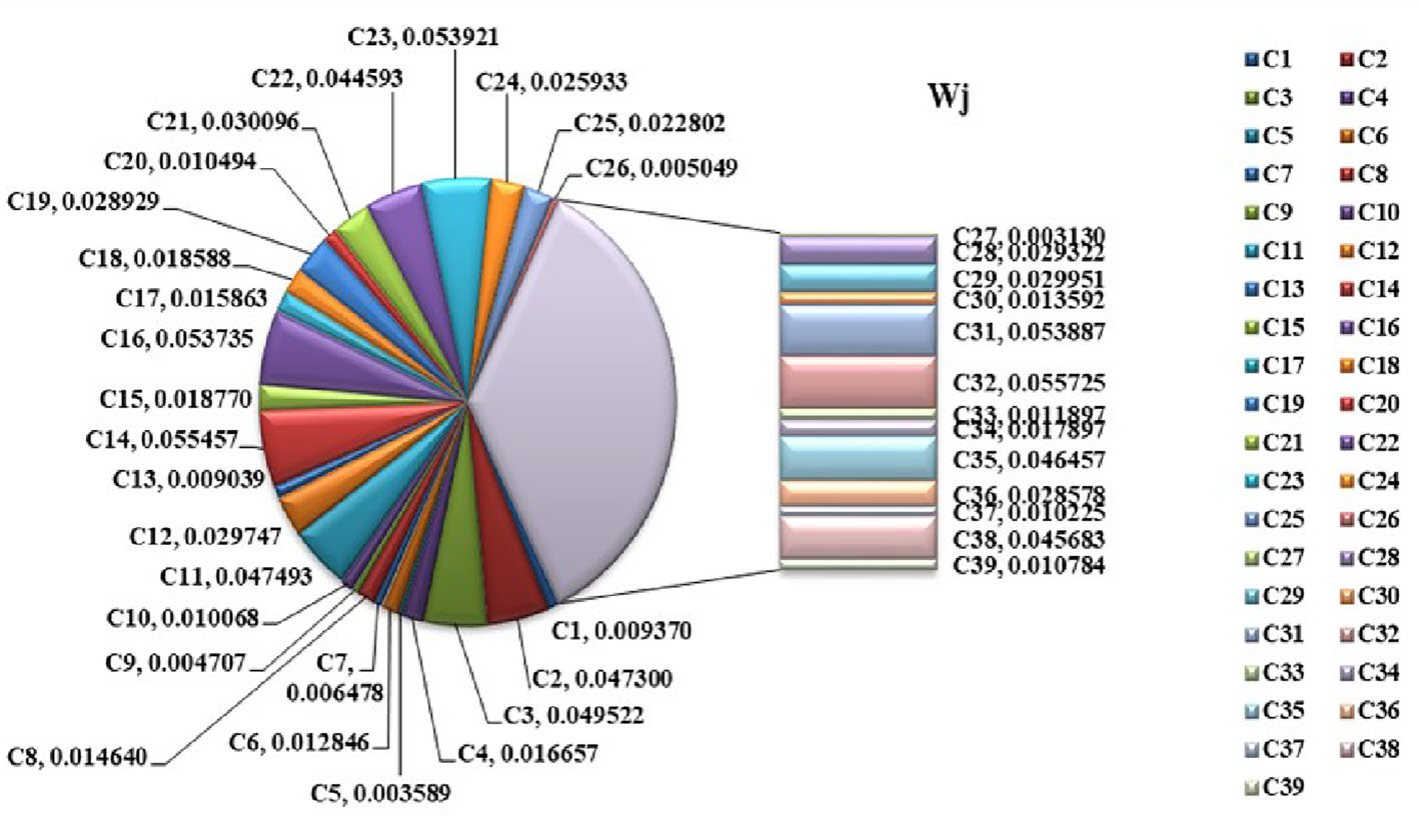
Representation of criteria weights.

**Figure 11:**
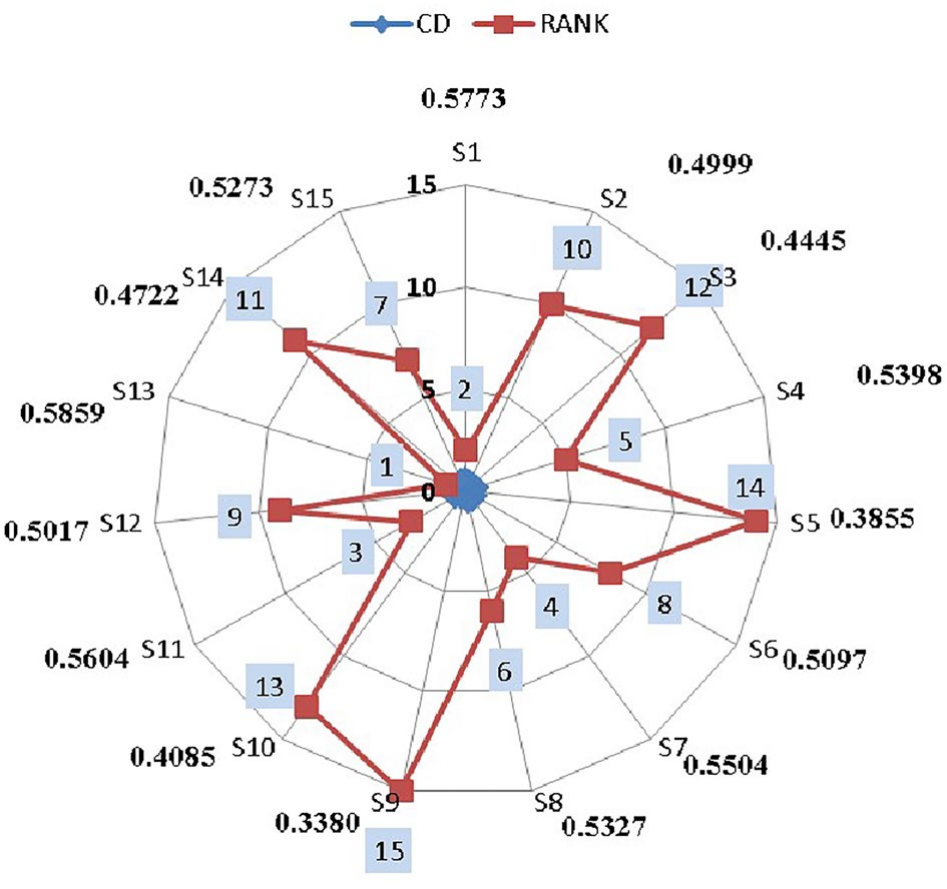
Visualization of supplier rankings.

## Results and discussion

The proposed approach incorporates IVTFN into the integrated TOPSIS-GRA method to select the most potential, resilient, and sustainable suppliers for pharmacies. The analysis carried out using real performance data of suppliers from three different retail pharmacies based on pioneering criteria which enhance the operational efficiency of pharmacies. Collectively formed data is converted into LDM using linguistic judgmental variables. Then LDM were transformed into an interval-valued triangular fuzzy matrix to handle the inherent uncertainty and vagueness of the linguistic data. Defuzzified the IVTFN using the CFCS algorithm to obtain crisp values, which were subsequently confined to the interval[0, 1] . Furthermore, the machine learning technique, SVR was leveraged to ascertain the prominent level of each criterion. After determining the weights, each element of the NDM was multiplied by its respective criterion weight to form the WNDM. The PIS and NIS were calculated, and GRA aided in determining the suppliers’ relative importance. The Grey Relational Grade (GRG) was calculated by multiplying the elements in the GRA matrix by the criterion weights and facilitated the determination of supplier rankings. The performance ranking of alternatives in numerical illustration $P_1$ showed $S_{7}>S_{5}>S_{2}>S_{1}>S_{6}>S_{3}>S_{4}$ indicating that $S_{7}$ is the most suitable and socially responsible supplier, followed by $S_{5}, S_{2}, S_{1}, S_{6}, S_{3}$ then by $S_{4}$. The numerical illustration of $P_2$ ranked the suppliers as $S_{3}>S_{1}>S_{4}>S_{5}>S_{7}>S_{11}>S_{10}>S_{8}>S_{6}>S_{9}>S_{2}$ concise that $S_{3}$ is highest followed by $S_{1}, S_{4}, S_{5}, S_{7}, S_{11}, S_{10}, S_{8}, S_{6}, S_{9}$, and $S_{2}$. The numerical illustration of $P_3$ shows $S_{13}>S_{1}>S_{11}>S_{7}>S_{4}>S_{8}>S_{15}>S_{6}>S_{12}>S_{2}>S_{14}>S_{3}>S_{10}>S_{5}>S_{9}$ whereas $S_{1}$ as the top supplier among 15 alternatives. Each illustration demonstrates the practical effectiveness of the model in ranking suppliers based on the supplier’s performance across various criteria. The varying results across the three illustrations further highlight the model’s adaptability to different contextual factors in supplier evaluation. If decision-makers adhere to these considerations and utilize the provided rankings, it will significantly improve the operational efficiency and sustainability practices of pharmacies, ultimately leading to better inventory management and ensuring the health and safety of the public.

## Practical and real-world implications

The proposed model offers a robust decision-making framework for supplier evaluation in pharmaceutical procurement. In today’s competitive landscape, effective cost management, sustainable procurement, and quality assurance depend on sourcing from reliable and high-performing suppliers. The integrated methodology systematically addresses both absolute and relational performance measures, reducing inconsistencies arising from human judgment and dynamic market conditions. By incorporating IVFNs, decision-makers can express assessments with greater flexibility, minimizing information loss and enhancing decision reliability. This methodology is highly applicable to real-world procurement scenarios, offering key advantages such as improved supplier selection accuracy, adaptability to market changes, enhanced risk mitigation, cross-industry applicability, and support for sustainable procurement. The model ensures precise supplier evaluation by considering both qualitative and quantitative factors, enabling organizations to identify the most reliable suppliers. Its ability to handle uncertain and imprecise data makes it effective in managing fluctuating supplier performance due to regulatory updates, demand shifts, and supply chain disruptions. Selection of an unsuitable supplier can lead to inefficiencies, increased costs, and operational risks. By combining fuzzy modeling techniques with a structured MCDM approach, this methodology enhances sustainable procurement efficiency, reduces uncertainty, supplier performances, and long-term supplier partnerships in dynamic environments. Moreover, the approach mitigates supplier-related risks such as delays, substandard product quality, and compliance failures, safeguarding patient safety and operational continuity. In a health sector where timely and reliable deliveries are essential, this model helps pharmaceuticals to minimize procurement inefficiencies, maintain an uninterrupted pharmaceutical supply and optimize inventory management.

## Comparative analyses

**Figure 12:**
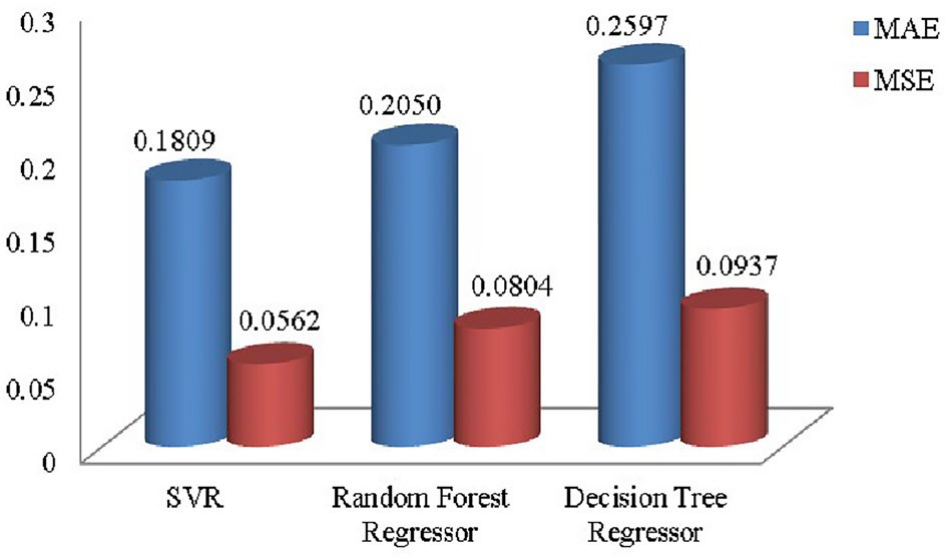
Weighted criteria values.

To assess the efficiency of the proposed FTOPSIS-GRA approach, the results were compared with the EDAS and ARAS methods. The comparative analysis revealed that while the leading supplier remained consistently ranked in all compared methods, ensuring reliability in decision-making, variations in evaluation scores highlighted differences in methodological sensitivity. The obtained supplier rankings are $S_{3}>S_{1}>S_{4}>S_{5}>S_{7}>S_{11}>S_{10}>S_{8}>S_{6}>S_{9}>S_{2}$ concise that $S_{3}$ is highest followed by $S_{1}, S_{4}, S_{5}, S_{7}, S_{11}, S_{10}, S_{8}, S_{6}, S_{9}$, and $S_{2}$; $S_{13}>S_{1}>S_{11}>S_{7}>S_{4}>S_{8}>S_{15}>S_{6}>S_{12}>S_{2}>S_{14}>S_{3}>S_{10}>S_{5}>S_{9}$ whereas $S_{1}$ is highest among 15 alternatives. Therefore, the proposed technique was effectively verified through comparative analysis. The results are presented in Tables 14 and 15, further validate the applicability and effectiveness of the proposed approach in dynamic supplier selection scenarios. In addition,the selection of SVR for criteria weight estimation was based on a comparative evaluation of machine learning techniques, including Random Forest Regressor and Decision Tree Regressor. The performance of these models was assessed using standard error metrics such as Mean Absolute Error (MAE) and Mean Squared Error (MSE). Among the tested models, SVR demonstrated the lowest MAE and MSE values, as shown in Fig. 12. It captures complex relationships within the data while minimizing error. Consequently, SVR was selected for weight determination.

**Table 14:** Comparison with EDAS method.

Suppliers	Proposed method		EDAS	
	Final score	RANK	Final score	RANK
$S_1$	0.6481	2	0.9439	2
$S_2$	0.3644	11	0.0737	11
$S_3$	0.6510	1	0.9606	1
$S_4$	0.6258	3	0.8376	3
$S_5$	0.5886	4	0.7558	4
$S_6$	0.4222	9	0.1709	9
$S_7$	0.5709	5	0.6220	5
$S_8$	0.4420	8	0.3434	8
$S_9$	0.3906	10	0.1255	10
$S_{10}$	0.4770	7	0.3935	7
$S_{11}$	0.4930	6	0.4344	6

**Table 15:** Comparison with ARAS method.

Suppliers	Proposed method		ARAS	
	Final score	RANK	Final score	RANK
$S_1$	0.5773	2	0.7626	2
$S_2$	0.4999	10	0.6720	10
$S_3$	0.4445	12	0.6111	12
$S_4$	0.5398	5	0.7378	5
$S_5$	0.3855	14	0.5510	14
$S_6$	0.5097	8	0.6963	8
$S_7$	0.5504	4	0.7457	4
$S_8$	0.5327	6	0.7234	6
$S_9$	0.3380	15	0.4964	15
$S_{10}$	0.4085	13	0.6003	13
$S_{11}$	0.5604	3	0.7504	3
$S_{12}$	0.5017	9	0.6751	9
$S_{13}$	0.5859	1	0.7887	1
$S_{14}$	0.4722	11	0.6622	11
$S_{15}$	0.5273	7	0.7215	7

## Sensitivity analysis

**Figure 13:**
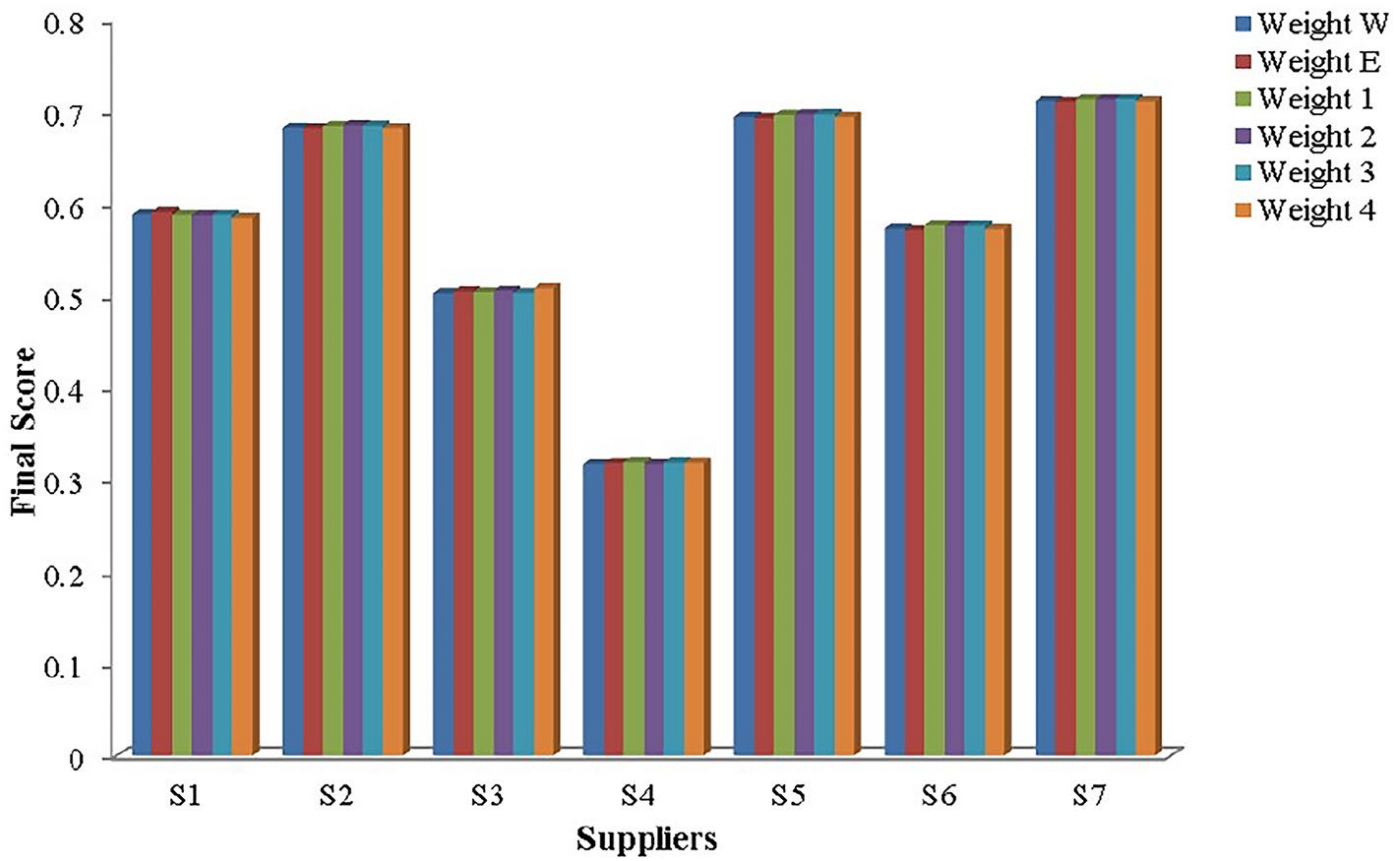
Sensitivity analysis of $P_1$ supplier ranking.

**Figure 14:**
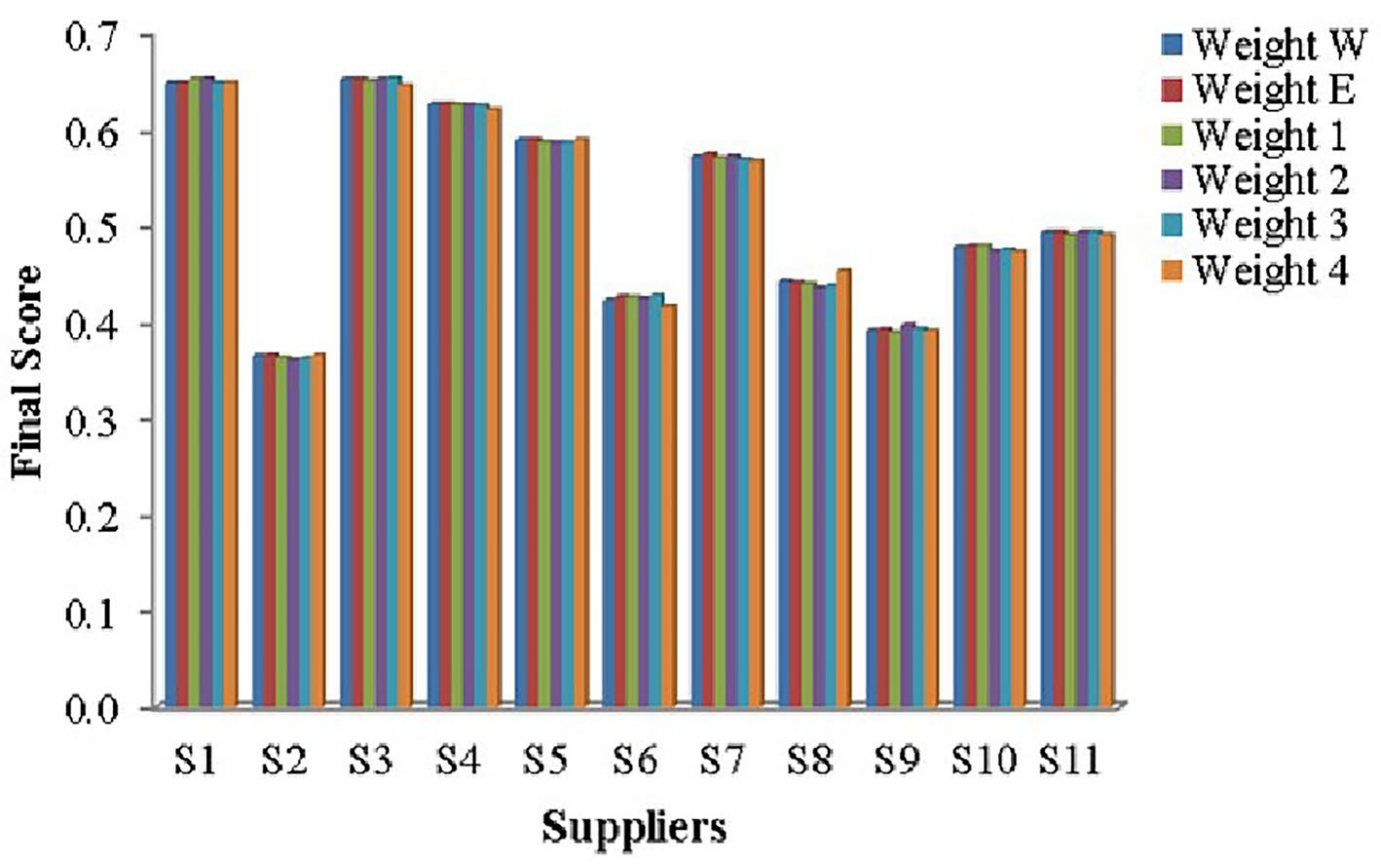
Sensitivity analysis of $P_2$ supplier ranking.

**Figure 15:**
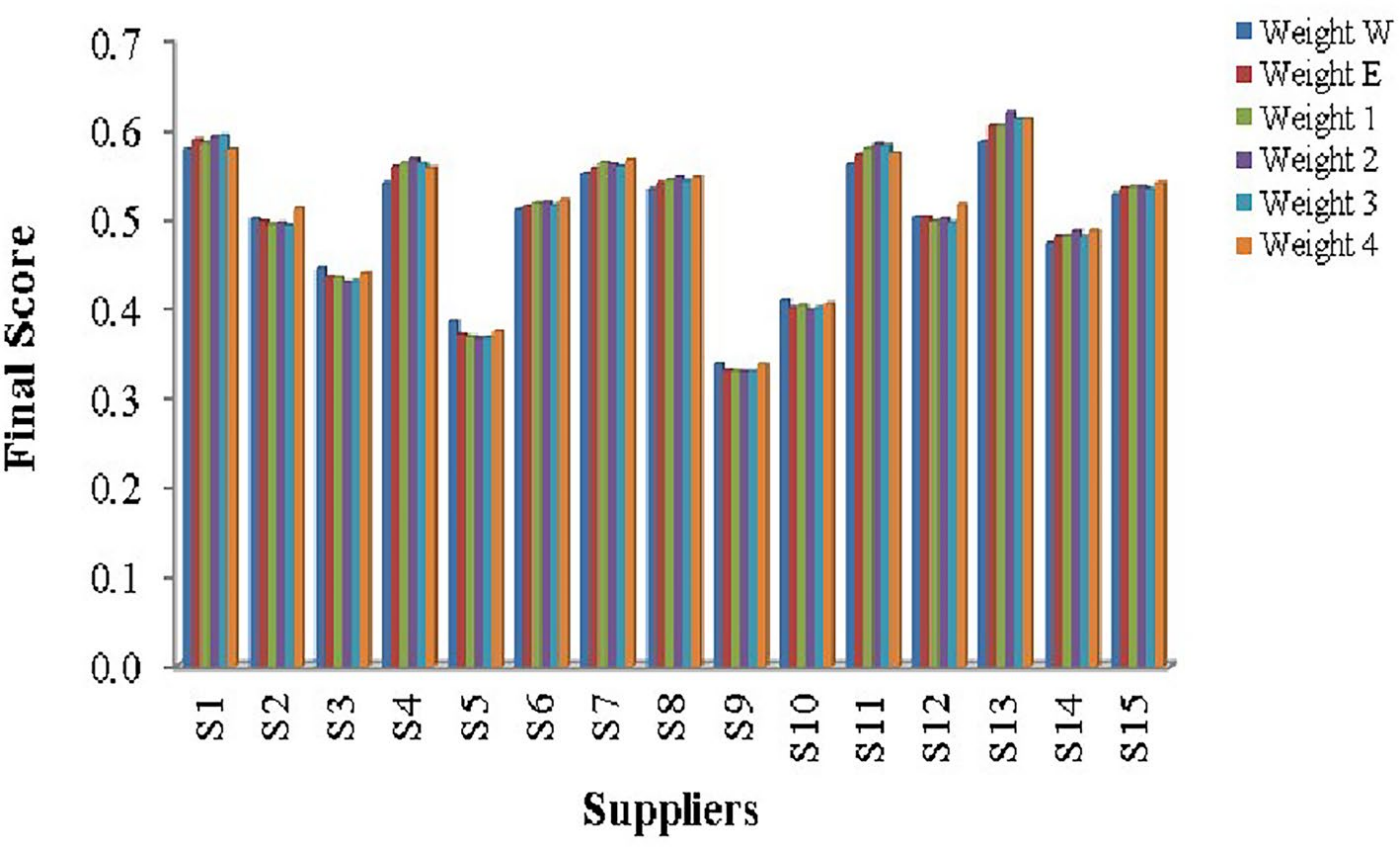
Sensitivity analysis of $P_3$ supplier ranking.

In MCDM approaches, sensitivity analysis is essential for verifying the stability and robustness of the proposed ranking against variations in weight parameters. The variation of weights for the sensitivity analysis is conducted based on decision experts’ suggestions, proposed SVR weights, and applied Entropy weights to assess the model’s stability. The computed weights were normalized to meet requirements of MCDM approach and used to reassess supplier rankings. The results confirm that despite fluctuations in weight parameters affecting the closeness coefficient values, the overall ranking remains unchanged. This demonstrates that the proposed decision-making model is stable and effective even in dynamic environments. Thus, Figs. 13, 14 and 15 presents the results of the sensitivity analysis.

## Conclusion

The proposed method successfully addresses the critical challenge of supplier selection in the pharmaceutical sector by proposing an innovative approach that integrates the FTOPSIS and GRA methods within a fuzzy environment. By employing the CFCS algorithm for defuzzification and Support Vector Regression (SVR) for weight assignment, this study has effectively transformed fuzzy data into crisp scores, thereby enhancing the accuracy and reliability of the supplier evaluation process. Through the implementation of this integrated methodology, three distinct supplier selection problems were solved, demonstrating its applicability and effectiveness in real-world scenarios. The results highlight the significance of employing advanced decision-making tools to ensure the timely availability of high-quality pharmaceutical products, ultimately contributing to improved health outcomes and safety.

## Future enhancements with potential limitations

The generalizability of this application is subject to certain limitations. Data was collected from three retail pharmacies, evaluating 33 suppliers through six decision experts, and converted into IVTF grades. Supplier selection is dynamic, with evolving criteria and emerging suppliers influenced by industry trends, regulations, and procurement policies. Findings are based on evaluations from retail pharmaceutical professionals. If data were sourced from hospitals or research institutions, variations in evaluation standards could lead to different outcomes. The criteria weights in this study were assigned using objective methods, specifically entropy and SVR. If subjective weighting were used instead, weight distributions might shift, impacting the results. Scalability is a key consideration, as applying this methodology to larger datasets with an increased number of suppliers and criteria may introduce complexities. Real-world testing under dynamic procurement conditions could further validate the model’s adaptability. Additionally, factors such as demand fluctuations, regulatory shifts, and supply chain disruptions may influence long-term supplier reliability. Future research can extend this supplier selection framework to broader sectors where procurement efficiency, transparency, and sustainability are critical. For instance, optimizing supplier evaluation for public healthcare systems could enhance the procurement of medicines, medical equipment, and emergency supplies, ensuring uninterrupted healthcare services. Fuzzy approaches can be explored to improve flexibility in diverse decision-making applications. Furthermore, the integration of advanced machine learning techniques and big data analytics could refine the supplier selection process, leading to more robust, resilient, and sustainable procurement practices.

## Supplementary Information

Supplementary Information.

## Data Availability

The data underlying the findings can be obtained from the corresponding author upon reasonable request.

## Abbreviations

AA: Above Average
AHP: Analytic Hierarchy Process
ANFIS: Adaptive Neuro-fuzzy inference system
ARAS: Additive Ratio Assessment
BA: Below Average
BCC: Banker–Charnes–Cooper
BWM: Best-Worst Method
CFCS: Converting Fuzzy data into Crisp Scores
CoCoSo: Combined Compromise Solution
CODAS: Combinative distance based assessment
CSFs: Critical Success Factors
DEA: Data Envelopment Analysis
DUEs: Dependent Uncertain Events
EDAS: Evaluation based on Distance from Average Solution
FPIS: Fuzzy Positive Ideal Solution
FAHP: Fuzzy Analytic Hierarchy Process
FNIS: Fuzzy Negative Ideal Solution
FST: Fuzzy Set Theory
FTOPSIS: Fuzzy Technique for Order Preference by Similarity to Ideal Solution
GA: Genetic Algorithm
GP: Genetic Programming
GRA: Grey Relational Analysis
GRC: Grey Relational Coefficient
IVFS: Interval-Valued Fuzzy Set
IVTFS: Interval-Valued Triangular Fuzzy Sets
IVTFN: Interval-Valued Triangular Fuzzy Number
LDM: Linguistic Decision Matrix
MADM: Multi-Attribute Decision-Making
MAE: Mean Absolute Error
MCDM: Multi-Criteria Decision-Making
MSE: Mean Squared Error
MILP: Mixed-Integer Linear Programming
MOMILP: Multi-objective mixed integer nonlinear programming
MOSO: Multi Ojective Stochastic Optimization
NDM: Normalized Decision Matrix
NS: Not Satisfactory
PCA: Principal Component Analysis
PROMETHEE: Preference ranking organization method for enrichment evaluation
PSO: Particle Swarm Optimization
QFD: Quality Function Deployment
SVR: Support Vector Regression
TFN: Triangular Fuzzy Number
TOPSIS: Technique for Order Preference by Similarity to Ideal Solution
TQM: Total Quality Management
WASPAS: Weighted aggregated sum product assessment
WNDM: Weighted Normalized Decision Matrix
