# Supplementary material for: Integrated MCDM framework for sustainable pharmacy supplier selection using pioneering criteria with fuzzy TOPSIS SVR and GRA
Source: Sci Rep. 2025 May 31;15:19144. doi: 10.1038/s41598-025-02975-z (PMC12126488; doi:10.1038/s41598-025-02975-z)
Supplement: Supplementary file 1 — Supplementary Information. [file 41598_2025_2975_MOESM1_ESM.pdf]

Supplementary Tables for numerical illustration

Supplementary Table 1. Defuzzified decision matrix

	S_1	S_2	S_3	S_4	S_5	S_6	S_7
C_1	0.5500	0.7500	0.5500	0.5500	0.8250	0.8250	0.5500
C_2	0.7500	0.6500	0.8250	0.7500	0.5500	0.6500	0.5500
C_3	0.1500	0.5500	0.1500	0.4500	0.7500	0.7500	0.7500
C_4	0.4500	0.8250	0.7500	0.7500	0.9000	0.8250	0.9000
C_5	0.7500	0.8250	0.8250	0.8250	0.9000	0.8250	0.9000
C_6	0.2500	0.5500	0.6500	0.2500	0.6500	0.6500	0.6500
C_7	0.6500	0.6500	0.6500	0.6500	0.7250	0.4500	0.3500
C_8	0.5500	0.5500	0.4500	0.5500	0.7250	0.5500	0.7250
C_9	0.6500	0.7500	0.8250	0.3500	0.7500	0.7500	0.6500
C_{10}	0.7500	0.9000	0.7500	0.8250	0.9000	0.9000	0.9000
C_{11}	0.9000	0.9000	0.6500	0.5500	0.9000	0.9000	0.9000
C_{12}	0.7500	0.8250	0.8250	0.8250	0.9000	0.9000	0.9000
C_{13}	0.6500	0.8250	0.8250	0.5500	0.8250	0.8250	0.7500
C_{14}	0.5500	0.7500	0.8250	0.5500	0.5500	0.5500	0.5500
C_{15}	0.7500	0.9000	0.8250	0.4500	0.9000	0.8250	0.9000
C_{16}	0.9000	0.9000	0.8250	0.7500	0.9000	0.8250	0.9000
C_{17}	0.5500	0.8250	0.6500	0.5500	0.7500	0.7500	0.7500
C_{18}	0.3500	0.5500	0.5500	0.4500	0.6500	0.6500	0.5500
C_{19}	0.4500	0.5500	0.5500	0.3500	0.7250	0.7250	0.7250
C_{20}	0.7500	0.7500	0.6500	0.4500	0.7500	0.7500	0.7500
C_{21}	0.4500	0.9000	0.4500	0.4500	0.8250	0.7500	0.8250
C_{22}	0.7500	0.7500	0.6500	0.6500	0.8250	0.9000	0.9000
C_{23}	0.5500	0.6500	0.7500	0.5500	0.8250	0.8250	0.8250
C_{24}	0.5500	0.7500	0.6500	0.5500	0.8250	0.8250	0.8250
C_{25}	0.6500	0.8250	0.8250	0.7500	0.8250	0.7500	0.8250
C_{26}	0.4500	0.6500	0.6500	0.4500	0.8250	0.9000	0.9000
C_{27}	0.8250	0.7500	0.7500	0.5500	0.7250	0.6500	0.7250
C_{28}	0.8250	0.9000	0.8250	0.7500	0.9000	0.9000	0.9000
C_{29}	0.7500	0.8250	0.7500	0.6500	0.8250	0.8250	0.8250
C_{30}	0.9000	0.9000	0.8250	0.7500	0.8250	0.8250	0.9000
C_{31}	0.6500	0.7500	0.5500	0.5500	0.8250	0.7500	0.6500
C_{32}	0.8250	0.8250	0.9000	0.9000	0.9000	0.9000	0.9000
C_{33}	0.5500	0.7500	0.6500	0.4500	0.7500	0.8250	0.7500
C_{34}	0.4500	0.8250	0.5500	0.6500	0.7250	0.6500	0.7250
C_{35}	0.4500	0.7250	0.6500	0.6500	0.8250	0.7500	0.7500
C_{36}	0.7445	0.8195	0.5445	0.7445	0.8946	0.8195	0.8195
C_{37}	0.1500	0.7500	0.3500	0.3500	0.8250	0.8250	0.8250
C_{38}	0.6250	0.6500	0.6500	0.5500	0.7500	0.5500	0.5500
C_{39}	0.5500	0.7500	0.5500	0.6500	0.9000	0.9000	0.9000

Supplementary Table 2. Normalized defuzzified decision matrix

	S_1	S_2	S_3	S_4	S_5	S_6	S_7
C_1	0.3107	0.4237	0.3107	0.3107	0.4660	0.4660	0.3107
C_2	0.4157	0.3602	0.4572	0.4157	0.3048	0.3602	0.3048
C_3	0.1003	0.3677	0.1003	0.3008	0.5014	0.5014	0.5014
C_4	0.2168	0.3974	0.3613	0.3613	0.4336	0.3974	0.4336
C_5	0.3386	0.3725	0.3725	0.3725	0.4064	0.3725	0.4064
C_6	0.1718	0.3780	0.4467	0.1718	0.4467	0.4467	0.4467
C_7	0.4078	0.4078	0.4078	0.4078	0.4548	0.2823	0.2196
C_8	0.3504	0.3504	0.2867	0.3504	0.4619	0.3504	0.4619
C_9	0.3559	0.4107	0.4517	0.1916	0.4107	0.4107	0.3559
C_{10}	0.3339	0.4007	0.3339	0.3673	0.4007	0.4007	0.4007
C_{11}	0.4119	0.4119	0.2975	0.2517	0.4119	0.4119	0.4119
C_{12}	0.3343	0.3677	0.3677	0.3677	0.4011	0.4011	0.4011
C_{13}	0.3246	0.4120	0.4120	0.2747	0.4120	0.4120	0.3745
C_{14}	0.3313	0.4518	0.4970	0.3313	0.3313	0.3313	0.3313
C_{15}	0.3514	0.4216	0.3865	0.2108	0.4216	0.3865	0.4216
C_{16}	0.3961	0.3961	0.3631	0.3300	0.3961	0.3631	0.3961
C_{17}	0.2985	0.4477	0.3527	0.2985	0.4070	0.4070	0.4070
C_{18}	0.2428	0.3816	0.3816	0.3122	0.4510	0.4510	0.3816
C_{19}	0.2842	0.3474	0.3474	0.2211	0.4579	0.4579	0.4579
C_{20}	0.4045	0.4045	0.3506	0.2427	0.4045	0.4045	0.4045
C_{21}	0.2462	0.4924	0.2462	0.2462	0.4513	0.4103	0.4513
C_{22}	0.3629	0.3629	0.3145	0.3145	0.3992	0.4355	0.4355
C_{23}	0.2747	0.3246	0.4120	0.3745	0.4120	0.4120	0.4120
C_{24}	0.2886	0.3935	0.3411	0.2886	0.4329	0.4329	0.4329
C_{25}	0.3146	0.3992	0.3992	0.3630	0.3992	0.3630	0.3992
C_{26}	0.2388	0.3450	0.3450	0.2388	0.4378	0.4776	0.4776
C_{27}	0.4359	0.3963	0.3963	0.2906	0.3831	0.3434	0.3831
C_{28}	0.3631	0.3961	0.3631	0.3300	0.3961	0.3961	0.3961
C_{29}	0.3630	0.3992	0.3630	0.3146	0.3992	0.3992	0.3992
C_{30}	0.4011	0.4011	0.3677	0.3343	0.3677	0.3677	0.4011
C_{31}	0.3602	0.4157	0.3048	0.3048	0.4572	0.4157	0.3602
C_{32}	0.3547	0.3547	0.3869	0.3869	0.3869	0.3869	0.3869
C_{33}	0.3030	0.4131	0.3581	0.2479	0.4131	0.4544	0.4131
C_{34}	0.2563	0.4699	0.3133	0.3703	0.4130	0.3703	0.4130
C_{35}	0.2448	0.3944	0.3536	0.3536	0.4488	0.4080	0.4080
C_{36}	0.3624	0.3989	0.2651	0.3624	0.4355	0.3989	0.3989
C_{37}	0.0885	0.4426	0.2065	0.2065	0.4868	0.4868	0.4868
C_{38}	0.3800	0.3952	0.3952	0.3344	0.4560	0.3344	0.3344
C_{39}	0.2743	0.3741	0.2743	0.3242	0.4489	0.4489	0.4489

Supplementary Table 3. Weighted normalized decision matrix

	S_1	S_2	S_3	S_4	S_5	S_6	S_7
C_1	0.0080	0.0109	0.0080	0.0080	0.0119	0.0119	0.0080
C_2	0.0107	0.0093	0.0118	0.0107	0.0079	0.0093	0.0079
C_3	0.0023	0.0086	0.0023	0.0070	0.0117	0.0117	0.0117
C_4	0.0056	0.0102	0.0093	0.0093	0.0111	0.0102	0.0111
C_5	0.0088	0.0097	0.0097	0.0097	0.0106	0.0097	0.0106
C_6	0.0043	0.0094	0.0111	0.0043	0.0111	0.0111	0.0111
C_7	0.0104	0.0104	0.0104	0.0104	0.0116	0.0072	0.0056
C_8	0.0090	0.0090	0.0074	0.0090	0.0119	0.0090	0.0119
C_9	0.0091	0.0105	0.0115	0.0049	0.0105	0.0105	0.0091
C_{10}	0.0087	0.0104	0.0087	0.0096	0.0104	0.0104	0.0104
C_{11}	0.0106	0.0106	0.0077	0.0065	0.0106	0.0106	0.0106
C_{12}	0.0087	0.0096	0.0096	0.0096	0.0104	0.0104	0.0104
C_{13}	0.0084	0.0107	0.0107	0.0071	0.0107	0.0107	0.0097
C_{14}	0.0085	0.0116	0.0128	0.0085	0.0085	0.0085	0.0085
C_{15}	0.0090	0.0108	0.0099	0.0054	0.0108	0.0099	0.0108
C_{16}	0.0103	0.0103	0.0095	0.0086	0.0103	0.0095	0.0103
C_{17}	0.0077	0.0116	0.0091	0.0077	0.0105	0.0105	0.0105
C_{18}	0.0062	0.0098	0.0098	0.0080	0.0116	0.0116	0.0098
C_{19}	0.0072	0.0088	0.0088	0.0056	0.0116	0.0116	0.0116
C_{20}	0.0104	0.0104	0.0090	0.0063	0.0104	0.0104	0.0104
C_{21}	0.0062	0.0124	0.0062	0.0062	0.0113	0.0103	0.0113
C_{22}	0.0094	0.0094	0.0081	0.0081	0.0103	0.0113	0.0113
C_{23}	0.0071	0.0084	0.0107	0.0097	0.0107	0.0107	0.0107
C_{24}	0.0074	0.0101	0.0088	0.0074	0.0111	0.0111	0.0111
C_{25}	0.0082	0.0104	0.0104	0.0094	0.0104	0.0104	0.0104
C_{26}	0.0060	0.0087	0.0087	0.0060	0.0111	0.0094	0.0121
C_{27}	0.0113	0.0103	0.0103	0.0075	0.0099	0.0089	0.0099
C_{28}	0.0095	0.0103	0.0095	0.0086	0.0103	0.0103	0.0103
C_{29}	0.0094	0.0104	0.0094	0.0082	0.0104	0.0104	0.0104
C_{30}	0.0104	0.0104	0.0096	0.0087	0.0104	0.0096	0.0096
C_{31}	0.0093	0.0107	0.0079	0.0079	0.0118	0.0107	0.0093
C_{32}	0.0092	0.0092	0.0101	0.0101	0.0101	0.0117	0.0101
C_{33}	0.0078	0.0106	0.0092	0.0064	0.0106	0.0106	0.0106
C_{34}	0.0066	0.0121	0.0081	0.0095	0.0106	0.0095	0.0106
C_{35}	0.0063	0.0102	0.0091	0.0091	0.0116	0.0105	0.0105
C_{36}	0.0094	0.0103	0.0069	0.0049	0.0113	0.0103	0.0103
C_{37}	0.0021	0.0105	0.0049	0.0087	0.0115	0.0115	0.0115
C_{38}	0.0099	0.0103	0.0103	0.0083	0.0118	0.0087	0.0087
C_{39}	0.0070	0.0096	0.0070	0.0083	0.0115	0.0115	0.0115

Supplementary Table 4. Defuzzified decision matrix

	S_1	S_2	S_3	S_4	S_5	S_6	S_7	S_8	S_9	S_{10}	S_{11}
C_1	0.1083	0.1959	0.3959	0.3959	0.3959	0.4835	0.1959	0.1959	0.4835	0.4835	0.1959
C_2	0.4835	0.4835	0.5835	0.6835	0.5835	0.4835	0.4835	0.4835	0.2835	0.3835	0.3835
C_3	0.4835	0.1959	0.5835	0.6835	0.3835	0.1959	0.4835	0.4835	0.1959	0.3835	0.5835
C_4	0.4835	0.2835	0.5835	0.4835	0.5835	0.5835	0.5835	0.3835	0.3835	0.4835	0.5835
C_5	0.5835	0.3835	0.6835	0.6835	0.4835	0.3835	0.3835	0.5835	0.4835	0.5835	0.5835
C_6	0.4835	0.2959	0.6835	0.7783	0.5835	0.1959	0.3835	0.5835	0.3835	0.3835	0.5835
C_7	0.5835	0.2959	0.4835	0.4835	0.3835	0.4835	0.4835	0.4835	0.3835	0.4835	0.4835
C_8	0.4835	0.2835	0.4835	0.4835	0.4835	0.3835	0.4835	0.2835	0.3835	0.3945	0.4835
C_9	0.5835	0.1959	0.5835	0.5835	0.5835	0.1959	0.2835	0.4835	0.4835	0.2835	0.3835
C_{10}	0.5840	0.3840	0.6840	0.5840	0.5840	0.4840	0.5840	0.5840	0.4840	0.4840	0.4840
C_{11}	0.5835	0.3835	0.6835	0.5835	0.3835	0.4835	0.5835	0.5835	0.2835	0.5835	0.3835
C_{12}	0.5835	0.4835	0.5835	0.5835	0.4835	0.5835	0.4835	0.3835	0.3835	0.5835	0.4835
C_{13}	0.4835	0.1959	0.6835	0.4835	0.5835	0.4835	0.4835	0.4835	0.3835	0.3835	0.5835
C_{14}	0.4835	0.1959	0.4835	0.4835	0.3835	0.3835	0.3835	0.4835	0.2835	0.3835	0.3835
C_{15}	0.5835	0.3835	0.5835	0.5835	0.4835	0.5835	0.5835	0.4835	0.4835	0.3835	0.3835
C_{16}	0.4835	0.3835	0.4835	0.5835	0.4835	0.3835	0.5835	0.3835	0.3835	0.3835	0.5835
C_{17}	0.4835	0.2835	0.4835	0.4835	0.4835	0.4835	0.4835	0.3835	0.4835	0.2835	0.4835
C_{18}	0.4835	0.1959	0.4835	0.6835	0.3835	0.3835	0.4835	0.4835	0.3835	0.3835	0.3835
C_{19}	0.5835	0.1083	0.6835	0.7783	0.5835	0.1959	0.3835	0.4835	0.1959	0.5835	0.3835
C_{20}	0.5835	0.2959	0.5835	0.6835	0.3835	0.1959	0.6835	0.2835	0.2835	0.3835	0.3835
C_{21}	0.5835	0.3835	0.4835	0.4835	0.5835	0.3835	0.3835	0.4835	0.3835	0.5835	0.2835
C_{22}	0.5835	0.4835	0.6835	0.4835	0.5835	0.4835	0.7783	0.5835	0.3835	0.3835	0.4835
C_{23}	0.7783	0.4835	0.6835	0.6835	0.6835	0.4835	0.6835	0.5835	0.2835	0.4835	0.2835
C_{24}	0.7783	0.3835	0.4835	0.5835	0.4835	0.2835	0.4835	0.3835	0.3835	0.3835	0.3835
C_{25}	0.6835	0.2959	0.4835	0.4835	0.4835	0.3835	0.3835	0.1959	0.3835	0.3835	0.3835
C_{26}	0.6835	0.1083	0.5835	0.3835	0.5835	0.3835	0.1955	0.4835	0.1959	0.3835	0.5835
C_{27}	0.5835	0.2959	0.5835	0.4835	0.4835	0.3835	0.3835	0.3835	0.3835	0.3835	0.3835
C_{28}	0.6835	0.5835	0.5835	0.5835	0.6835	0.4835	0.7778	0.4835	0.4835	0.5835	0.4835
C_{29}	0.6835	0.2959	0.4835	0.3835	0.5835	0.1956	0.5835	0.3835	0.3835	0.3835	0.3835
C_{30}	0.5835	0.3835	0.4835	0.4835	0.5835	0.2835	0.5835	0.3835	0.3835	0.3835	0.3835
C_{31}	0.4835	0.4835	0.5835	0.3835	0.5835	0.3835	0.4835	0.3835	0.1959	0.5835	0.2835
C_{32}	0.6835	0.3835	0.4835	0.4835	0.4835	0.3835	0.5835	0.2835	0.3835	0.5835	0.3835
C_{33}	0.4835	0.1959	0.4835	0.4835	0.3835	0.2835	0.4835	0.2835	0.3835	0.3835	0.3835
C_{34}	0.3835	0.2835	0.5835	0.4835	0.4835	0.2835	0.4835	0.1959	0.3835	0.3835	0.2835
C_{35}	0.3835	0.2835	0.5835	0.4835	0.4835	0.2835	0.1959	0.1959	0.1959	0.1959	0.2835
C_{36}	0.3835	0.2959	0.5835	0.4835	0.3835	0.2835	0.1959	0.2835	0.1959	0.2835	0.2835
C_{37}	0.5835	0.1959	0.5835	0.3835	0.5835	0.2835	0.3835	0.2835	0.4835	0.3835	0.2835
C_{38}	0.4835	0.4904	0.6835	0.3956	0.6835	0.4835	0.4835	0.5835	0.3835	0.3835	0.3835
C_{39}	0.6835	0.5835	0.6835	0.7779	0.6835	0.6835	0.7778	0.6835	0.3835	0.7779	0.7783

Supplementary Table 5. Normalization of decision matrix

	S_1	S_2	S_3	S_4	S_5	S_6	S_7	S_8	S_9	S_{10}	S_{11}
C_1	0.0936	0.1694	0.3424	0.3424	0.3424	0.4182	0.1694	0.1694	0.4182	0.4182	0.1694
C_2	0.2947	0.2947	0.3557	0.4166	0.3557	0.2947	0.2947	0.2947	0.1728	0.2338	0.2338
C_3	0.3217	0.1303	0.3882	0.4548	0.2552	0.1303	0.3217	0.3217	0.1303	0.2552	0.3882
C_4	0.2901	0.1701	0.3501	0.2901	0.3501	0.3501	0.3501	0.2301	0.2301	0.2901	0.3501
C_5	0.3259	0.2142	0.3818	0.3818	0.2701	0.2142	0.2142	0.3259	0.2701	0.3259	0.3259
C_6	0.2842	0.1739	0.4017	0.4574	0.3429	0.1151	0.2254	0.3429	0.2254	0.2254	0.3429
C_7	0.3799	0.1927	0.3148	0.3148	0.2497	0.3148	0.3148	0.3148	0.2497	0.3148	0.3148
C_8	0.3408	0.1998	0.3408	0.3408	0.3408	0.2703	0.3408	0.1998	0.2703	0.2780	0.3408
C_9	0.3921	0.1316	0.3921	0.3921	0.3921	0.1316	0.1905	0.3249	0.3249	0.1905	0.2577
C_{10}	0.3236	0.2128	0.3790	0.3236	0.3236	0.2682	0.3236	0.3236	0.2682	0.2682	0.2682
C_{11}	0.3412	0.2242	0.3996	0.3412	0.2242	0.2827	0.3412	0.3412	0.1658	0.3412	0.2242
C_{12}	0.3408	0.2824	0.3408	0.3408	0.2824	0.3408	0.2824	0.2240	0.2240	0.3408	0.2824
C_{13}	0.2970	0.1203	0.4199	0.2970	0.3585	0.2970	0.2970	0.2970	0.2356	0.2356	0.3585
C_{14}	0.3615	0.1464	0.3615	0.3615	0.2867	0.2867	0.2867	0.3615	0.2120	0.2867	0.2867
C_{15}	0.3459	0.2274	0.3459	0.3459	0.2867	0.3459	0.3459	0.2867	0.2867	0.2274	0.2274
C_{16}	0.3084	0.2446	0.3084	0.3722	0.3084	0.2446	0.3722	0.2446	0.2446	0.2446	0.3722
C_{17}	0.3276	0.1921	0.3276	0.3276	0.3276	0.3276	0.3276	0.2599	0.3276	0.1921	0.3276
C_{18}	0.3278	0.1328	0.3278	0.4633	0.2600	0.2600	0.3278	0.3278	0.2600	0.2600	0.2600
C_{19}	0.3544	0.0658	0.4151	0.4727	0.3544	0.1190	0.2329	0.2937	0.1190	0.3544	0.2329
C_{20}	0.3812	0.1933	0.3812	0.4465	0.2505	0.1280	0.4465	0.1852	0.1852	0.2505	0.2505
C_{21}	0.3773	0.2480	0.3127	0.3127	0.3773	0.2480	0.2480	0.3127	0.2480	0.3773	0.1833
C_{22}	0.3201	0.2652	0.3749	0.2652	0.3201	0.2652	0.4269	0.3201	0.2104	0.2104	0.2652
C_{23}	0.4059	0.2521	0.3564	0.3564	0.3564	0.2521	0.3564	0.3043	0.1479	0.2521	0.1479
C_{24}	0.4959	0.2444	0.3081	0.3718	0.3081	0.1806	0.3081	0.2444	0.2444	0.2444	0.2444
C_{25}	0.4797	0.2077	0.3394	0.3394	0.3394	0.2692	0.2692	0.1375	0.2692	0.2692	0.2692
C_{26}	0.4555	0.0721	0.3888	0.2556	0.3888	0.2556	0.1305	0.3222	0.1305	0.3222	0.3888
C_{27}	0.4009	0.2104	0.4009	0.3319	0.3319	0.2627	0.2627	0.2627	0.2610	0.3104	0.2603
C_{28}	0.3489	0.3104	0.2977	0.2977	0.3487	0.2469	0.3969	0.2467	0.2455	0.3104	0.2501
C_{29}	0.4556	0.2100	0.3232	0.2556	0.3889	0.1310	0.3891	0.2556	0.2556	0.3001	0.2561
C_{30}	0.3839	0.2501	0.3178	0.3178	0.3838	0.1867	0.3839	0.2529	0.2501	0.3104	0.2504
C_{31}	0.3210	0.3210	0.3856	0.2538	0.3859	0.2539	0.3201	0.2539	0.1301	0.4001	0.1900
C_{32}	0.4309	0.2390	0.3049	0.3049	0.3049	0.2419	0.3678	0.1789	0.2400	0.4103	0.2390
C_{33}	0.3678	0.1501	0.3678	0.3678	0.2906	0.2159	0.3679	0.2160	0.2891	0.3001	0.2891
C_{34}	0.2887	0.2101	0.4381	0.3638	0.3636	0.2135	0.3636	0.1478	0.2881	0.3001	0.2081
C_{35}	0.3291	0.2400	0.5019	0.4159	0.4159	0.2441	0.1687	0.1687	0.1691	0.2002	0.2391
C_{36}	0.3291	0.2451	0.5009	0.4149	0.3301	0.2439	0.1679	0.2439	0.1701	0.2104	0.2391
C_{37}	0.4149	0.4139	0.4148	0.2728	0.4149	0.2019	0.2731	0.2020	0.3401	0.3001	0.2001
C_{38}	0.2878	0.2890	0.4067	0.2359	0.4068	0.2880	0.2880	0.3478	0.2301	0.2001	0.2205
C_{39}	0.2977	0.2491	0.2978	0.3410	0.2978	0.2978	0.3401	0.2978	0.1681	0.3001	0.3391

Supplementary Table 6. Decision matrix with weighted normalization

	S_1	S_2	S_3	S_4	S_5	S_6	S_7	S_8	S_9	S_{10}	S_{11}
C_1	0.0023	0.0041	0.0084	0.0084	0.0084	0.0102	0.0041	0.0041	0.0102	0.0102	0.0041
C_2	0.0077	0.0077	0.0092	0.0108	0.0092	0.0077	0.0077	0.0077	0.0045	0.0061	0.0061
C_3	0.0080	0.0032	0.0096	0.0113	0.0063	0.0032	0.0080	0.0080	0.0032	0.0063	0.0096
C_4	0.0076	0.0044	0.0091	0.0076	0.0091	0.0091	0.0091	0.0060	0.0060	0.0076	0.0091
C_5	0.0085	0.0056	0.0099	0.0099	0.0070	0.0056	0.0056	0.0085	0.0070	0.0085	0.0085
C_6	0.0071	0.0044	0.0101	0.0115	0.0086	0.0029	0.0057	0.0086	0.0057	0.0057	0.0086
C_7	0.0100	0.0051	0.0083	0.0083	0.0066	0.0083	0.0083	0.0083	0.0066	0.0083	0.0083
C_8	0.0089	0.0052	0.0089	0.0089	0.0089	0.0071	0.0089	0.0052	0.0071	0.0073	0.0089
C_9	0.0098	0.0033	0.0098	0.0098	0.0098	0.0033	0.0048	0.0081	0.0081	0.0048	0.0064
C_{10}	0.0085	0.0056	0.0100	0.0085	0.0085	0.0071	0.0085	0.0085	0.0071	0.0071	0.0071
C_{11}	0.0088	0.0058	0.0103	0.0088	0.0058	0.0073	0.0088	0.0088	0.0043	0.0088	0.0058
C_{12}	0.0090	0.0074	0.0090	0.0090	0.0074	0.0090	0.0074	0.0059	0.0059	0.0090	0.0074
C_{13}	0.0077	0.0031	0.0108	0.0077	0.0092	0.0077	0.0077	0.0077	0.0061	0.0061	0.0092
C_{14}	0.0094	0.0038	0.0094	0.0094	0.0074	0.0074	0.0074	0.0094	0.0055	0.0074	0.0074
C_{15}	0.0091	0.0060	0.0091	0.0091	0.0075	0.0091	0.0091	0.0075	0.0075	0.0060	0.0060
C_{16}	0.0081	0.0064	0.0081	0.0097	0.0081	0.0064	0.0097	0.0064	0.0064	0.0064	0.0097
C_{17}	0.0086	0.0050	0.0086	0.0086	0.0086	0.0086	0.0086	0.0068	0.0086	0.0050	0.0086
C_{18}	0.0084	0.0034	0.0084	0.0119	0.0067	0.0067	0.0084	0.0084	0.0067	0.0067	0.0067
C_{19}	0.0086	0.0016	0.0100	0.0114	0.0086	0.0029	0.0056	0.0071	0.0029	0.0086	0.0056
C_{20}	0.0095	0.0048	0.0095	0.0111	0.0062	0.0032	0.0111	0.0046	0.0046	0.0062	0.0062
C_{21}	0.0098	0.0065	0.0081	0.0081	0.0098	0.0065	0.0065	0.0081	0.0065	0.0098	0.0048
C_{22}	0.0083	0.0069	0.0097	0.0069	0.0083	0.0069	0.0111	0.0083	0.0055	0.0055	0.0069
C_{23}	0.0104	0.0064	0.0091	0.0091	0.0091	0.0064	0.0091	0.0078	0.0038	0.0064	0.0038
C_{24}	0.0127	0.0063	0.0079	0.0095	0.0079	0.0046	0.0079	0.0063	0.0063	0.0063	0.0063
C_{25}	0.0123	0.0053	0.0087	0.0087	0.0087	0.0069	0.0069	0.0035	0.0069	0.0069	0.0069
C_{26}	0.0111	0.0018	0.0095	0.0062	0.0095	0.0062	0.0032	0.0079	0.0032	0.0062	0.0095
C_{27}	0.0095	0.0053	0.0095	0.0009	0.0009	0.0069	0.0069	0.0069	0.0069	0.0069	0.0069
C_{28}	0.0086	0.0078	0.0080	0.0080	0.0086	0.0060	0.0104	0.0065	0.0065	0.0079	0.0065
C_{29}	0.0119	0.0050	0.0079	0.0061	0.0104	0.0032	0.0104	0.0065	0.0065	0.0061	0.0065
C_{30}	0.0094	0.0066	0.0079	0.0080	0.0104	0.0052	0.0104	0.0066	0.0066	0.0069	0.0066
C_{31}	0.0082	0.0081	0.0104	0.0059	0.0104	0.0056	0.0082	0.0064	0.0033	0.0104	0.0048
C_{32}	0.0110	0.0062	0.0081	0.0081	0.0080	0.0058	0.0104	0.0046	0.0062	0.0101	0.0062
C_{33}	0.0104	0.0039	0.0104	0.0104	0.0083	0.0059	0.0101	0.0056	0.0075	0.0083	0.0075
C_{34}	0.0069	0.0055	0.0110	0.0089	0.0089	0.0051	0.0089	0.0038	0.0074	0.0069	0.0055
C_{35}	0.0079	0.0058	0.0012	0.0104	0.0104	0.0058	0.0036	0.0042	0.0042	0.0037	0.0059
C_{36}	0.0079	0.0064	0.0130	0.0104	0.0076	0.0058	0.0036	0.0062	0.0042	0.0058	0.0062
C_{37}	0.0101	0.0035	0.0101	0.0068	0.0101	0.0046	0.0065	0.0051	0.0087	0.0068	0.0051
C_{38}	0.0068	0.0076	0.0110	0.0058	0.0110	0.0068	0.0068	0.0089	0.0059	0.0061	0.0059
C_{39}	0.0081	0.0067	0.0079	0.0089	0.0081	0.0081	0.0089	0.0078	0.0044	0.0091	0.0089

Supplementary Table 7. Defuzzified decision matrix

	S_1	S_2	S_3	S_4	S_5	S_6	S_7	S_8	S_9	S_{10}	S_{11}	S_{12}	S_{13}	S_{14}	S_{15}
C_1	0.472	0.372	0.572	0.572	0.472	0.572	0.572	0.472	0.472	0.472	0.572	0.274	0.472	0.472	0.472
C_2	0.472	0.185	0.372	0.772	0.272	0.572	0.672	0.372	0.372	0.572	0.272	0.272	0.472	0.285	0.272
C_3	0.385	0.372	0.572	0.372	0.472	0.372	0.285	0.372	0.274	0.472	0.185	0.285	0.272	0.185	0.272
C_4	0.485	0.285	0.185	0.772	0.272	0.672	0.672	0.672	0.272	0.672	0.872	0.672	0.772	0.672	0.672
C_5	0.672	0.185	0.185	0.872	0.372	0.572	0.672	0.672	0.472	0.385	0.772	0.572	0.672	0.572	0.672
C_6	0.185	0.372	0.272	0.099	0.272	0.185	0.285	0.099	0.099	0.185	0.185	0.385	0.185	0.272	0.272
C_7	0.185	0.472	0.372	0.772	0.472	0.472	0.572	0.672	0.372	0.372	0.572	0.472	0.185	0.672	0.572
C_8	0.772	0.272	0.272	0.772	0.472	0.572	0.472	0.672	0.272	0.472	0.672	0.472	0.472	0.672	0.572
C_9	0.472	0.572	0.272	0.672	0.472	0.572	0.672	0.772	0.372	0.372	0.772	0.572	0.772	0.572	0.572
C_{10}	0.772	0.572	0.468	0.772	0.472	0.672	0.772	0.772	0.272	0.472	0.872	0.572	0.772	0.572	0.672
C_{11}	0.772	0.372	0.472	0.572	0.472	0.672	0.672	0.672	0.272	0.572	0.772	0.372	0.672	0.672	0.672
C_{12}	0.672	0.572	0.472	0.772	0.372	0.672	0.672	0.672	0.372	0.572	0.672	0.372	0.772	0.572	0.672
C_{13}	0.672	0.672	0.472	0.772	0.472	0.772	0.472	0.572	0.272	0.372	0.560	0.572	0.772	0.672	0.672
C_{14}	0.385	0.572	0.372	0.272	0.485	0.572	0.472	0.472	0.372	0.372	0.185	0.272	0.372	0.472	0.572
C_{15}	0.772	0.672	0.572	0.672	0.472	0.572	0.672	0.672	0.472	0.572	0.772	0.572	0.772	0.572	0.672
C_{16}	0.872	0.572	0.772	0.572	0.472	0.672	0.772	0.772	0.372	0.472	0.772	0.472	0.872	0.572	0.772
C_{17}	0.772	0.672	0.572	0.572	0.472	0.572	0.472	0.572	0.472	0.572	0.772	0.572	0.872	0.672	0.572
C_{18}	0.285	0.372	0.372	0.472	0.472	0.472	0.472	0.372	0.372	0.472	0.372	0.372	0.372	0.472	0.572
C_{19}	0.472	0.572	0.772	0.185	0.285	0.099	0.372	0.472	0.185	0.472	0.472	0.385	0.185	0.099	0.272
C_{20}	0.285	0.572	0.472	0.472	0.472	0.472	0.472	0.472	0.472	0.372	0.672	0.372	0.472	0.472	0.472
C_{21}	0.372	0.572	0.472	0.472	0.472	0.472	0.472	0.372	0.472	0.472	0.572	0.572	0.472	0.472	0.472
C_{22}	0.672	0.374	0.472	0.472	0.472	0.472	0.472	0.472	0.372	0.472	0.672	0.572	0.472	0.472	0.472
C_{23}	0.472	0.672	0.672	0.672	0.372	0.672	0.872	0.872	0.572	0.872	0.572	0.572	0.672	0.572	0.772
C_{24}	0.772	0.572	0.572	0.772	0.472	0.672	0.772	0.872	0.472	0.472	0.672	0.672	0.772	0.672	0.772
C_{25}	0.772	0.672	0.472	0.772	0.472	0.672	0.672	0.672	0.472	0.572	0.672	0.572	0.872	0.672	0.672
C_{26}	0.672	0.385	0.185	0.572	0.272	0.572	0.572	0.572	0.272	0.472	0.772	0.572	0.872	0.672	0.472
C_{27}	0.672	0.472	0.372	0.672	0.185	0.572	0.572	0.472	0.272	0.472	0.672	0.572	0.672	0.572	0.472
C_{28}	0.472	0.772	0.272	0.772	0.472	0.672	0.872	0.672	0.472	0.472	0.772	0.672	0.672	0.572	0.872
C_{29}	0.772	0.572	0.285	0.772	0.372	0.572	0.672	0.572	0.372	0.372	0.672	0.572	0.672	0.672	0.772
C_{30}	0.572	0.672	0.572	0.772	0.472	0.672	0.672	0.672	0.472	0.472	0.672	0.572	0.672	0.672	0.772
C_{31}	0.672	0.572	0.472	0.672	0.285	0.472	0.572	0.572	0.272	0.272	0.572	0.672	0.572	0.472	0.572
C_{32}	0.872	0.672	0.672	0.672	0.472	0.672	0.772	0.772	0.472	0.472	0.872	0.572	0.872	0.672	0.672
C_{33}	0.472	0.572	0.472	0.572	0.472	0.572	0.572	0.572	0.372	0.372	0.572	0.572	0.872	0.472	0.572
C_{34}	0.472	0.472	0.572	0.772	0.472	0.672	0.672	0.472	0.472	0.372	0.572	0.572	0.772	0.472	0.572
C_{35}	0.572	0.372	0.372	0.672	0.272	0.672	0.772	0.772	0.272	0.185	0.672	0.572	0.772	0.472	0.572
C_{36}	0.872	0.472	0.572	0.472	0.472	0.472	0.572	0.472	0.472	0.472	0.572	0.472	0.472	0.472	0.572
C_{37}	0.572	0.572	0.472	0.672	0.472	0.472	0.572	0.472	0.472	0.285	0.385	0.672	0.672	0.672	0.572
C_{38}	0.572	0.472	0.672	0.272	0.572	0.372	0.372	0.372	0.472	0.372	0.285	0.472	0.272	0.272	0.272
C_{39}	0.672	0.672	0.772	0.572	0.472	0.672	0.872	0.872	0.372	0.572	0.572	0.572	0.372	0.472	0.772

Supplementary Table 8. Normalized decision matrix

	S_1	S_2	S_3	S_4	S_5	S_6	S_7	S_8	S_9	S_{10}	S_{11}	S_{12}	S_{13}	S_{14}	S_{15}
C_1	0.248	0.195	0.300	0.300	0.248	0.300	0.300	0.248	0.248	0.248	0.295	0.144	0.248	0.248	0.248
C_2	0.274	0.107	0.216	0.448	0.158	0.332	0.390	0.216	0.216	0.332	0.158	0.158	0.274	0.165	0.158
C_3	0.277	0.268	0.412	0.268	0.340	0.268	0.205	0.268	0.197	0.338	0.133	0.205	0.196	0.133	0.196
C_4	0.205	0.120	0.078	0.326	0.115	0.284	0.284	0.284	0.115	0.284	0.368	0.284	0.326	0.284	0.284
C_5	0.295	0.081	0.081	0.383	0.164	0.251	0.295	0.295	0.208	0.169	0.339	0.251	0.295	0.251	0.295
C_6	0.199	0.400	0.293	0.106	0.293	0.199	0.307	0.106	0.106	0.199	0.199	0.414	0.199	0.293	0.293
C_7	0.094	0.240	0.190	0.393	0.240	0.240	0.291	0.342	0.190	0.189	0.291	0.236	0.094	0.342	0.291
C_8	0.362	0.128	0.128	0.362	0.222	0.269	0.222	0.315	0.128	0.222	0.315	0.222	0.222	0.315	0.269
C_9	0.209	0.253	0.120	0.297	0.209	0.253	0.297	0.341	0.164	0.164	0.341	0.253	0.341	0.253	0.253
C_{10}	0.306	0.227	0.185	0.306	0.187	0.266	0.306	0.306	0.108	0.187	0.346	0.227	0.306	0.227	0.266
C_{11}	0.334	0.161	0.204	0.247	0.204	0.290	0.290	0.290	0.118	0.247	0.334	0.161	0.290	0.290	0.290
C_{12}	0.286	0.243	0.201	0.329	0.158	0.286	0.286	0.286	0.158	0.243	0.286	0.158	0.329	0.243	0.286
C_{13}	0.288	0.288	0.202	0.331	0.202	0.331	0.202	0.245	0.117	0.159	0.239	0.245	0.331	0.288	0.288
C_{14}	0.231	0.344	0.224	0.164	0.291	0.344	0.284	0.284	0.224	0.224	0.206	0.295	0.295	0.265	0.211
C_{15}	0.312	0.271	0.231	0.271	0.191	0.231	0.271	0.271	0.191	0.231	0.312	0.231	0.312	0.231	0.271
C_{16}	0.336	0.220	0.297	0.220	0.182	0.259	0.297	0.297	0.143	0.182	0.297	0.182	0.336	0.219	0.297
C_{17}	0.320	0.279	0.237	0.237	0.196	0.237	0.196	0.237	0.196	0.237	0.319	0.237	0.362	0.279	0.237
C_{18}	0.173	0.226	0.226	0.286	0.286	0.286	0.286	0.226	0.226	0.286	0.226	0.226	0.226	0.286	0.347
C_{19}	0.306	0.371	0.500	0.120	0.185	0.064	0.241	0.306	0.120	0.306	0.306	0.249	0.119	0.064	0.176
C_{20}	0.155	0.312	0.257	0.257	0.257	0.257	0.257	0.257	0.257	0.203	0.366	0.203	0.257	0.257	0.257
C_{21}	0.199	0.306	0.253	0.253	0.253	0.253	0.253	0.199	0.253	0.253	0.306	0.306	0.253	0.253	0.253
C_{22}	0.348	0.193	0.244	0.244	0.244	0.244	0.244	0.244	0.193	0.244	0.348	0.296	0.244	0.244	0.244
C_{23}	0.181	0.258	0.258	0.258	0.143	0.258	0.334	0.334	0.219	0.334	0.219	0.219	0.258	0.219	0.296
C_{24}	0.295	0.218	0.218	0.295	0.179	0.256	0.295	0.333	0.179	0.179	0.256	0.256	0.295	0.256	0.295
C_{25}	0.304	0.265	0.186	0.304	0.186	0.265	0.265	0.265	0.186	0.225	0.265	0.225	0.344	0.265	0.265
C_{26}	0.311	0.178	0.086	0.265	0.126	0.265	0.265	0.265	0.126	0.218	0.357	0.265	0.403	0.311	0.218
C_{27}	0.326	0.229	0.179	0.326	0.089	0.277	0.277	0.229	0.132	0.229	0.326	0.277	0.326	0.277	0.229
C_{28}	0.187	0.305	0.108	0.305	0.187	0.265	0.344	0.265	0.187	0.187	0.305	0.265	0.265	0.226	0.344
C_{29}	0.341	0.253	0.126	0.341	0.164	0.253	0.297	0.253	0.164	0.164	0.297	0.253	0.297	0.297	0.253
C_{30}	0.233	0.274	0.233	0.315	0.193	0.274	0.274	0.274	0.193	0.193	0.274	0.233	0.274	0.274	0.315
C_{31}	0.327	0.279	0.229	0.327	0.139	0.229	0.279	0.279	0.133	0.133	0.279	0.327	0.279	0.229	0.279
C_{32}	0.325	0.251	0.251	0.251	0.176	0.251	0.288	0.288	0.176	0.176	0.325	0.214	0.325	0.251	0.251
C_{33}	0.221	0.268	0.221	0.268	0.221	0.268	0.268	0.268	0.175	0.175	0.268	0.268	0.409	0.221	0.268
C_{34}	0.214	0.214	0.259	0.349	0.214	0.304	0.304	0.214	0.214	0.168	0.259	0.259	0.349	0.214	0.259
C_{35}	0.258	0.168	0.168	0.303	0.123	0.303	0.348	0.348	0.123	0.083	0.303	0.258	0.348	0.213	0.303
C_{36}	0.421	0.228	0.276	0.228	0.228	0.228	0.276	0.228	0.228	0.228	0.276	0.228	0.228	0.228	0.276
C_{37}	0.271	0.271	0.224	0.318	0.224	0.224	0.271	0.224	0.224	0.135	0.182	0.318	0.318	0.318	0.271
C_{38}	0.348	0.287	0.408	0.165	0.348	0.226	0.226	0.226	0.287	0.226	0.173	0.287	0.165	0.165	0.165
C_{39}	0.272	0.272	0.313	0.232	0.191	0.272	0.353	0.353	0.151	0.232	0.232	0.232	0.151	0.191	0.313

Supplementary Table 9. Weighted normalised decision matrix

	S_1	S_2	S_3	S_4	S_5	S_6	S_7	S_8	S_9	S_{10}	S_{11}	S_{12}	S_{13}	S_{14}	S_{15}
C_1	0.0023	0.0018	0.0028	0.0028	0.0023	0.0028	0.0028	0.0023	0.0023	0.0023	0.0028	0.0013	0.0023	0.0023	0.0023
C_2	0.0129	0.0051	0.0102	0.0212	0.0075	0.0157	0.0184	0.0102	0.0102	0.0157	0.0075	0.0075	0.0129	0.0078	0.0075
C_3	0.0137	0.0133	0.0204	0.0133	0.0168	0.0133	0.0102	0.0133	0.0098	0.0168	0.0066	0.0102	0.0097	0.0066	0.0010
C_4	0.0034	0.0020	0.0013	0.0054	0.0019	0.0047	0.0047	0.0047	0.0019	0.0047	0.0061	0.0047	0.0054	0.0047	0.0047
C_5	0.0011	0.0003	0.0003	0.0014	0.0006	0.0009	0.0011	0.0011	0.0007	0.0006	0.0012	0.0009	0.0011	0.0009	0.0009
C_6	0.0026	0.0051	0.0038	0.0014	0.0038	0.0026	0.0039	0.0014	0.0014	0.0026	0.0026	0.0053	0.0026	0.0038	0.0038
C_7	0.0006	0.0016	0.0012	0.0025	0.0016	0.0016	0.0019	0.0022	0.0012	0.0012	0.0019	0.0016	0.0006	0.0022	0.0022
C_8	0.0053	0.0019	0.0019	0.0053	0.0032	0.0039	0.0032	0.0046	0.0019	0.0032	0.0046	0.0032	0.0032	0.0046	0.0046
C_9	0.0010	0.0012	0.0006	0.0014	0.0010	0.0012	0.0014	0.0016	0.0008	0.0008	0.0016	0.0012	0.0016	0.0012	0.0012
C_{10}	0.0031	0.0023	0.0019	0.0031	0.0019	0.0027	0.0031	0.0031	0.0011	0.0019	0.0035	0.0023	0.0031	0.0023	0.0023
C_{11}	0.0158	0.0076	0.0097	0.0117	0.0097	0.0138	0.0138	0.0138	0.0056	0.0117	0.0158	0.0076	0.0138	0.0138	0.0138
C_{12}	0.0085	0.0072	0.0060	0.0098	0.0047	0.0085	0.0085	0.0085	0.0047	0.0072	0.0085	0.0047	0.0098	0.0072	0.0085
C_{13}	0.0026	0.0026	0.0018	0.0030	0.0018	0.0030	0.0018	0.0022	0.0011	0.0014	0.0022	0.0022	0.0030	0.0026	0.0026
C_{14}	0.0128	0.0191	0.0124	0.0091	0.0162	0.0191	0.0157	0.0157	0.0124	0.0124	0.0062	0.0091	0.0124	0.0157	0.019
C_{15}	0.0059	0.0051	0.0043	0.0051	0.0036	0.0043	0.0051	0.0051	0.0036	0.0038	0.0059	0.0043	0.0059	0.0043	0.0046
C_{16}	0.0180	0.0118	0.0160	0.0118	0.0098	0.0139	0.0160	0.0160	0.0077	0.0098	0.0160	0.0098	0.0180	0.0118	0.016
C_{17}	0.0051	0.0044	0.0038	0.0038	0.0031	0.0038	0.0031	0.0038	0.0031	0.0038	0.0051	0.0038	0.0057	0.0044	0.0044
C_{18}	0.0032	0.0042	0.0042	0.0053	0.0053	0.0053	0.0053	0.0042	0.0042	0.0053	0.0042	0.0038	0.0037	0.0053	0.0056
C_{19}	0.0089	0.0107	0.0145	0.0035	0.0053	0.0019	0.0070	0.0089	0.0035	0.0089	0.0089	0.0072	0.0035	0.0019	0.0049
C_{20}	0.0016	0.0033	0.0027	0.0027	0.0027	0.0027	0.0027	0.0027	0.0027	0.0021	0.0038	0.0023	0.0028	0.0033	0.0033
C_{21}	0.0056	0.0088	0.0079	0.0078	0.0076	0.0077	0.0075	0.0060	0.0080	0.0081	0.0091	0.0090	0.0079	0.0082	0.0083
C_{22}	0.0155	0.0088	0.0111	0.0111	0.0110	0.0110	0.0110	0.0110	0.0088	0.0110	0.0146	0.0128	0.0109	0.0109	0.0109
C_{23}	0.0098	0.0136	0.0137	0.0137	0.0078	0.0139	0.0177	0.0179	0.0119	0.0177	0.0120	0.0119	0.0139	0.0118	0.0156
C_{24}	0.0076	0.0056	0.0056	0.0078	0.0053	0.0068	0.0075	0.0089	0.0050	0.0048	0.0067	0.0068	0.0075	0.0067	0.0083
C_{25}	0.0069	0.0060	0.0036	0.0066	0.0036	0.0060	0.0060	0.0060	0.0036	0.00460	0.0060	0.0045	0.0075	0.0055	0.0061
C_{26}	0.0016	0.0009	0.0004	0.0009	0.0006	0.0009	0.0009	0.0009	0.0006	0.0009	0.0017	0.0009	0.0015	0.0017	0.0009
C_{27}	0.0009	0.0007	0.0006	0.0009	0.0003	0.0009	0.0009	0.0007	0.0004	0.0007	0.0009	0.0009	0.0009	0.0009	0.0007
C_{28}	0.0055	0.0089	0.0029	0.0087	0.0049	0.0079	0.0111	0.0079	0.0046	0.0047	0.0089	0.0078	0.0079	0.0067	0.0111
C_{29}	0.0102	0.0075	0.0035	0.0111	0.0046	0.0083	0.0086	0.0082	0.0046	0.0046	0.0088	0.0075	0.0086	0.0086	0.0078
C_{30}	0.0032	0.0037	0.0029	0.0035	0.0029	0.0035	0.0035	0.0039	0.0025	0.0025	0.0035	0.0034	0.0035	0.0037	0.0035
C_{31}	0.0176	0.0146	0.0119	0.0179	0.0066	0.0119	0.0146	0.0146	0.0069	0.0069	0.0149	0.0176	0.0149	0.0116	0.0149
C_{32}	0.0181	0.0138	0.0136	0.0136	0.0111	0.0136	0.0156	0.0156	0.0111	0.0111	0.0178	0.0116	0.0176	0.0138	0.0138
C_{33}	0.0026	0.0026	0.0026	0.0027	0.0026	0.0027	0.0026	0.0026	0.0017	0.0016	0.0027	0.0026	0.0046	0.0027	0.0026
C_{34}	0.0038	0.0038	0.0047	0.0056	0.0038	0.0049	0.0049	0.0041	0.0041	0.0029	0.0048	0.0047	0.0057	0.0041	0.0051
C_{35}	0.0118	0.0078	0.0078	0.0138	0.0058	0.0138	0.0158	0.0158	0.0058	0.0038	0.0138	0.0118	0.0158	0.0011	0.0138
C_{36}	0.0115	0.0067	0.0075	0.0067	0.0067	0.0065	0.0079	0.0065	0.0065	0.0067	0.0075	0.0067	0.0067	0.0067	0.0081
C_{37}	0.0028	0.0027	0.0017	0.0027	0.0017	0.0017	0.0027	0.0017	0.0017	0.0007	0.0017	0.0027	0.0027	0.0027	0.0027
C_{38}	0.0159	0.0129	0.0189	0.0076	0.0156	0.0111	0.0111	0.0111	0.0129	0.0111	0.0076	0.0129	0.0076	0.0076	0.0079
C_{39}	0.0029	0.0029	0.0029	0.0019	0.0019	0.0029	0.0039	0.0039	0.0022	0.0022	0.0022	0.0022	0.0020	0.0020	0.0032
